# Bifunctional Carbosilane Dendrimers for the Design of Multipurpose Hydrogels with Antibacterial Action

**DOI:** 10.1021/acs.chemmater.3c02027

**Published:** 2023-12-26

**Authors:** Silvia Muñoz-Sánchez, Irene Heredero-Bermejo, Francisco Javier de la Mata, Sandra García-Gallego

**Affiliations:** † University of Alcala, Department of Organic and Inorganic Chemistry and Research Institute in Chemistry “Andrés M. Del Río” (IQAR), 28805 Madrid, Spain; ‡ University of Alcala, Department of Biomedicine and Biotechnology, 28805 Madrid, Spain; § Networking Research Center on Bioengineering, Biomaterials and Nanomedicine (CIBER-BBN), 28029 Madrid, Spain; ∥ Institute Ramón y Cajal for Health Research (IRYCIS), 28034 Madrid, Spain

## Abstract

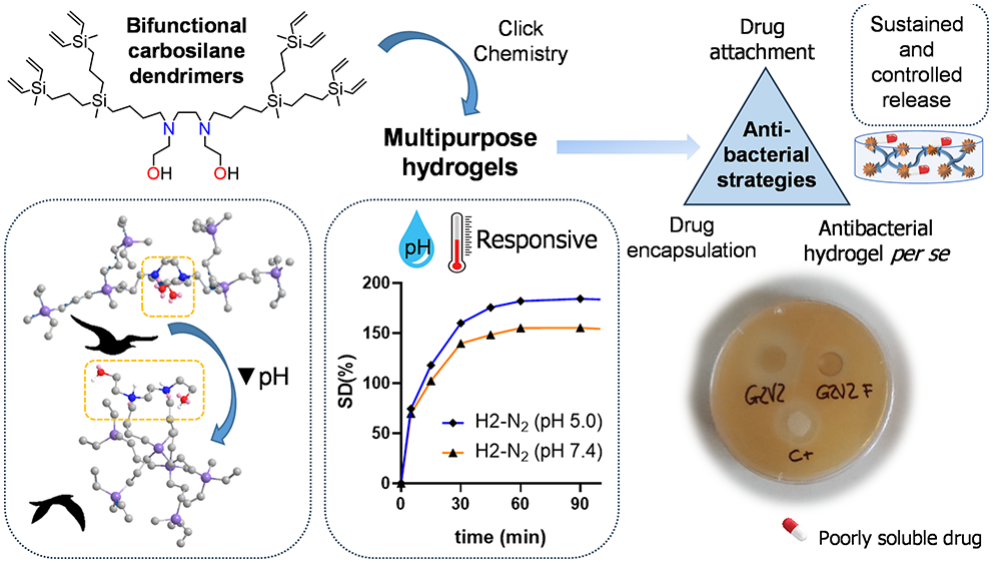

The emergence of antibiotic resistance is a serious global health problem. There is an incessant demand for new antimicrobial drugs and materials that can address this global issue from different angles. Dendritic hydrogels have appeared as a promising strategy. A family of bifunctional amphiphilic carbosilane dendrimers was designed and employed as nanosized cross-linking points for the synthesis of high-swelling hydrogels using the highly efficient Thiol–Ene *click* reaction for their preparation. Both stoichiometric and off-stoichiometric conditions were studied, generating hydrogels with pendant hydroxyl or alkene moieties. These hydrogels were found to be tunable antibacterial materials. They can easily be postmodified with relevant antibiotic moieties through covalent attachment on the hydroxyl or alkene pendant groups, generating ammonium-decorated networks with temperature and pH-responsive properties. Additionally, they can efficiently encapsulate drugs with poor solubility in water, like ciprofloxacin, and perform a sustained release over time, as demonstrated in preliminary assays against *Staphylococcus aureus*.

## 1 Introduction

In the last decades, many infections have been suppressed or eradicated due to the increase in public hygiene and the development of biomedical technology.^1^ However, the rapid emergence of antimicrobial resistance (AMR) in pathogenic microbes is now a global health problem, causing over 5 million deaths associated with bacterial AMR per year.^2^ Due to this problem, and along with the solubility, cytotoxicity, and overdose problems caused by conventional antibiotics,^3^ researchers are encouraged to study new antibacterial approaches to meet the incessant demand for new drugs and materials.^4^ For example, natural extracts from plants and animals have received broad attention, including polyphenols, terpenoids, essential oils, and polypeptides.^3^ Metals like silver, zinc, copper, iron, and gold have also been employed as antimicrobial systems for centuries, with different mechanisms of action.^5^ More recently, other materials have been in the spotlight, such as antimicrobial polymers,^6^ antimicrobial peptides,^7^ metal nanoparticles,^5^ and hydrogels.^8,9^

Hydrogels are three-dimensional, polymeric, hydrophilic networks capable of absorbing large amounts of water or biological fluids without dissolving. Due to this capacity and along with porosity and soft consistency,^10^ they have a great physicochemical similarity to the native extracellular matrix and, therefore, high biocompatibility.^11^ Hydrogel design and preparation play a very important role in the final application, and lead to several commercial uses like contact lenses, hygiene products, wound dressings, tissue engineering, and drug delivery.^12^ Hydrogels may also exhibit drastic volume changes in response to specific stimuli, such as temperature, light, or pH, which can be employed, for example, to control drug release.

Despite these advantages, hydrogels also have some limitations. For example, reversible hydrogels usually show a limited mechanical strength and are prone to permanent breakage. Sometimes the high water content and large pore sizes result in a relatively rapid unwanted drug release. Besides, although some hydrogels are sufficiently deformable to be injectable, others require surgical implantation for certain applications.^13^ Additionally, most hydrogels present hydrophilic networks, being poorly compatible with hydrophobic drugs and thus becoming inefficient reservoirs for these types of drugs. Therefore, an improvement in the properties of these hydrogels would lead to an optimization of their potential.^11^

The incorporation of nanosized dendritic molecules in the hydrogel structure can improve its properties, which can be tuned by altering the generation, scaffold, solubility, or end groups of the dendritic molecule.^14^ Dendrimers are highly branched and monodisperse molecules composed of three structural components: a core, repeating branching units, and various peripheral functional groups.^15^ Due to these repeating branching units, their growth is exponential, providing very distinctive properties compared to linear polymers. Furthermore, these macromolecules adopt a globular conformation and are synthesized in a controlled manner, thus enabling control of the size, the architecture, and the reactive end groups. Due to all these characteristics, dendrimers can be used as cross-linking points for the design of innovative networks, providing a perfect synthetic control over the structure-to-activity.

Surprisingly, only few examples of antibacterial dendritic hydrogels have been reported in the literature, which can be classified as (i) hydrogels containing inorganic nanoparticles; (ii) hydrogels containing antibacterial drugs; (iii) hydrogels with inherent antibacterial capacities; and (iv) hydrogels with a synergistic effect.^9^ For example, Navath et al. examined the formation of biodegradable hydrogels obtained by cross-linking thiopyridyl functionalized G4-PAMAM dendrimer with 8-arm polyethylene glycol (PEG) by disulfide bridging. The formation of the disulfide bonds is essential for a slower release of the drug, due to the decreased pore size.^16^ McMahon et al. prepared a hydrogel using a hyperbranched PEGDAPEGMEMA copolymer and thiol-modified hyaluronic acid as a cross-linker by a thiol–ene reaction encapsulating silver sulfadiazine in the copolymer system to create an antimicrobial wound care dressing improving antibacterial activity.^17^ Malkoch et al. developed a hydrogel based on polyester systems with amino groups that prevented bacterial infections for 24 h in the absence of antibiotics. The polyester scaffold provided multivalency and hydrolytic degradability and the cross-linker offered solubility and biocompatibility.^18^

Unlike the previous examples, based on hydrophilic dendritic scaffolds, our group recently reported the first dendritic hydrogels using carbosilane dendrimers as cross-linking points.^19^ Carbosilane dendrimers present silicon–carbon (Si–C) bonds in their structure providing to the macromolecule great kinetic stability, high flexibility, and very low polarity.^20,21^ Multiple examples have been presented on the activity of carbosilane dendrimers as promising antiviral, antibacterial, and antiparasitic agents, where the hydrophilic–lipophilic balance exhibits a crucial role in the antimicrobial activity.^20^ In this work, we designed the first family of bifunctional carbosilane dendrimers. The multivalence and perfect structure of these dendrimers have been exploited to generate amphiphilic hydrogels, with potential as new antibacterial materials. The preparation of these hydrogels was carried out by click chemistry through the highly efficient thiol–ene coupling (TEC) reaction.^22,23^ These networks can be tuned as multipurpose materials with antibacterial properties, as demonstrated below.

## 2 Experimental Section

Comprehensive details of the materials and methods used in this work are described in Supporting Information. Synthetic protocols toward dendrimers **1**–**3** and all hydrogels are described below. Structure and purity of **1**–**3** were confirmed via ^1^H, ^13^C, and 2D-NMR using a Bruker Neo400 spectrometer, elemental analysis, and MALDI-TOF spectrometry. Hydrogels were cured using a UV darkroom Vilber CN-15.LC (30 W at 365 nm) and characterized through their swelling degree (SD%), cross-linking degree (CD%), and RAMAN-confocal microscopy (Thermo Scientific DXR). The loading and release of cargo from the hydrogels were explored via FT-IR and HPLC (Agilent 1200). *In vitro* antibacterial activity was measured through an inhibition zone assay on *S. aureus* and scanning electron microscopy.

### 2.1 Materials

Reagents and solvents were purchased from commercial sources and used as received. Vinyl-decorated dendrons BrGnV_m_ (**I**–**III**) were synthesized as previously reported.^10^

### 2.2 Synthesis and Characterization of Carbosilane Dendrimers

#### 2.2.1 General Procedure for Dendrimers **1**–**3**

The corresponding precursor dendron (BrG1V_2_ (**I**), BrG2V_4_ (**II**), or BrG3V_8_ (**III**) (2 equiv),^22^*N*,*N*′-bis(2-hydroxyethyl) ethylenediamine (1 equiv), K_2_CO_3_ (3 equiv), and NaI (2 equiv) were added in a stirring flask with the minimum amount of acetone at 90 °C. Once the reaction is finished after 4–6 h, the solution is filtered, and the solvent is evaporated. Then, the dendrimers are purified by size exclusion chromatography in acetone. The resultant dendrimers **1**–**3** were isolated as yellow oils in 75% yield.

#### 2.2.2 Dendrimer **1**

C_24_H_48_N_2_O_2_Si_2_ (452.83 g/mol). Yellowish oil soluble in chloroform. ^1^H NMR (400 MHz CDCl_3_): δ 6.05–5.66 (12 H, m, C*H*C*H*_*2*_), 3.55 (4 H, t, C*H*_*2*_OH), 2.55 (4 H, t, N–C*H*_*2*_CH_2_–OH), 2.52 (4 H, s, N–C*H*_*2*_C*H*_*2*_–N), 2.45 (4 H, m, N–C*H*_*2*_CH_2_CH_2_CH_2_–Si), 1.46 (4 H, m, N–CH_2_C*H*_*2*_CH_2_CH_2_–Si), 1.27 (4 H, m, N–CH_2_CH_2_C*H*_*2*_CH_2_–Si), 0.63 (4 H, t, N–CH_2_CH_2_CH_2_C*H*_*2*_-Si), 0.08 (6 H, s, Si–C*H*_*3*_(vinyl)). ^13^C NMR (400 MHz CDCl_3_): δ 136.82 (*C*HCH_2_), 132.97 (CH*C*H_2_), 60.06 (*C*H_2_OH), 56.03 (N–*C*H_2_CH_2_–OH), 55.36 (N–*C*H_2_CH_2_CH_2_CH_2_–Si), 52.62 (N–*C*H_2_*C*H_2_–N), 30.18 (N–CH_2_*C*H_2_CH_2_CH_2_–Si), 21.64 (N–CH_2_CH_2_*C*H_2_CH_2_–Si), 14.06 (N–CH_2_CH_2_CH_2_*C*H_2_–Si), −5.36 (Si(*C*H)_3_). Elemental analysis: theo.: C, 63.66; H, 10.68; N, 6.19. Exp.: C, 63.64; H, 10.61; N, 6.34. *m*/*z* 452.33. Exp. 453.4 (M + H^+^).

#### 2.2.3 Dendrimer **2**

C_48_H_96_N_2_O_2_Si_6_ (901.82 g/mol). Yellowish oil soluble in chloroform. ^1^H NMR (400 MHz CDCl_3_): δ 6.04–5.68 (24 H, m, C*H*C*H*_*2*_), 3.59 (4 H, t, C*H*_*2*_OH), 2.59 (4 H, t, N–C*H*_*2*_CH_2_–OH), 2.57 (4 H, s, N–C*H*_*2*_C*H*_*2*_–N), 2.48 (4 H, m, N–C*H*_*2*_CH_2_CH_2_CH_2_–Si), 1.46 (4 H, m, N–CH_2_C*H*_*2*_CH_2_CH_2_–Si), 1.32 (8 H, m, Si–CH_2_CH_2_CH_2_–Si), 1.21 (4 H, m, N–CH_2_CH_2_C*H*_*2*_CH_2_–Si), 0.68 (8 H, t, Si–CH_2_CH_2_C*H*_*2*_–Si), 0.54 (8 H, t, Si–*CH*_*2*_CH_2_CH_2_–Si), 0.45 (4 H, t, N–CH_2_CH_2_CH_2_C*H*_*2*_–Si), 0.10 (12 H, s, Si–C*H*_*3*_(vinyl)), −0.12 (6 H, s, SiC*H*_*3*_). ^13^C-RMN (400 MHz CDCl_3_): δ 137.15 (CH = CH_2_), 132.68 (CH = CH_2_), 60.19 (*C*H_2_OH), 55.96 (N–*C*H_2_CH_2_–OH), 55.56 (N-*C*H_2_CH_2_CH_2_CH_2_–Si), 52.76 (N(*C*H_2_)_2_N), 30.52 (N–CH_2_*C*H_2_CH_2_CH_2_–Si), 22.02 (N–CH_2_CH_2_*C*H_2_CH_2_–Si), 18.74 (Si-*C*H_2_CH_2_CH_2_–Si), 18.54 (Si-CH_2_CH_2_*C*H_2_–Si), 18.31 (Si-CH_2_*C*H_2_CH_2_–Si), 14.06 (N–CH_2_CH_2_CH_2_*C*H_2_–Si), −5.09 (Si(*C*H)_3_), −5.19 (Si(*C*H)_3_(vinyl)). Elemental analysis: theo.: C, 63.93; H, 10.73; N, 3.11. Exp.: C, 63.40; H, 9.54; N, 6.13. *m*/*z* 900.61; Exp. 901.6 (M + H^+^).

#### 2.2.4 Dendrimer **3**

C_96_H_192_N_2_O_2_Si_14_ (1799.79 g/mol). Yellowish oil soluble in chloroform. ^1^H NMR (400 MHz CDCl_3_): δ 6.04–5.67 (48 H, m, C*H*C*H*_*2*_), 3.58 (4 H, t, C*H*_*2*_OH), 2.59 (4 H, t, N–C*H*_*2*_CH_2_–OH), 2.57 (4 H, s, N–C*H*_*2*_C*H*_*2*_–N), 2.48 (4 H, m, N–C*H*_*2*_CH_2_CH_2_CH_2_–Si), 1.47 (4 H, m, N–CH_2_C*H*_*2*_CH_2_CH_2_–Si), 1.31 (28H, m, Si–CH_2_CH_2_CH_2_–Si, N–CH_2_CH_2_C*H*_*2*_CH_2_–Si), 0.69 (16 H, m, Si–CH_2_CH_2_C*H*_*2*_–Si–(vinyl)), 0.53 (36 H, m, N–CH_2_CH_2_CH_2_C*H*_*2*_–Si, Si–*CH*_*2*_CH_2_C*H*_*2*_–Si, Si–C*H*_*2*_CH_2_CH_2_–Si–(vinyl)), 0.11 (24 H, s, Si–C*H*_*3*_(vinyl)), −0.11 (18 H, s, SiC*H*_*3*_). ^13^C-RMN (400 MHz CDCl_3_): δ 137.46 (*C*HCH_2_), 132.76 (CH*C*H_2_), 60.53 (*C*H_2_OH), 56.11 (N–*C*H_2_CH_2_–OH), 56.00 (N–*C*H_2_CH_2_CH_2_CH_2_–Si), 52.97 (N(*C*H_2_)_2_N), 30.37 (N–CH_2_*C*H_2_CH_2_CH_2_–Si), 22.14 (N–CH_2_CH_2_*C*H_2_CH_2_–Si), 19.12–18.48 ((Si–*C*H_2_CH_2_CH_2_–Si, Si–CH_2_CH_2_*C*H_2_–Si, Si–CH_2_*C*H_2_CH_2_–Si, Si–*C*H_2_CH_2_CH_2_–Si(vinyl), Si–CH_2_CH_2_*C*H_2_–Si(vinyl), Si–CH_2_*C*H_2_CH_2_–Si(vinyl)), 14.26 (N–CH_2_CH_2_CH_2_*C*H_2_–Si), −4.81 (Si(*C*H)_3_), −5.12 (Si(*C*H)_3_(vinyl)). Elemental analysis: theo.: C, 64.07; H, 10.75; N, 1.56. Exp.: C, 63.89; H, 9.98; N, 1.47. *m*/*z* 1799.18; Exp. 1800.1 (M + H^+^).

### 2.3 Synthetic Procedure for Dendritic Hydrogels

#### 2.3.1 Hydrogels Synthesis

PEG1k(SH)_2_ (2 eq. for Hy[(Si-G0V_4_)x(P)]; 1.5 eq. for Hy[(Si-G0V_3_)x(P)]V_1_; 2 eq. for Hy[(N_2_O_2_-G1V_4_)x(P)] and Hy[(N_2_O_2_-G2V_4_)x(P)]V_4_; 3 eq. for Hy[(N_2_O_2_-G2V_6_)x(P)]V_2_; 4 eq. for Hy[(N_2_O_2_-G2V_8_)x(P)]; and 8 eq. for Hy[(N_2_O_2_-G3V_16_)x(P)] were weighed into one vial and a mixture of THF:MeOH (1:2) was added. Next, the corresponding dendrimer (1 equiv) and DMPA (5 mol % of vinyl groups) were added. The resulting mixture was introduced into several Teflon plugs with a capacity of around 200 μL. Hydrogels were cured using a UV lamp (30 W at 365 nm) for the required time described in Table 1. Then, the hydrogels were washed several times with acetone until DMPA was eliminated, as confirmed by TLC (hexane:ethyl acetate, 80:20).

**Table 1:** Summary of Selected Parameters of N_2_O_2_-Core Dendrimers **1**–**3** and Si-Core Dendrimers **4**–**6**, as well as the Derived Dendritic Hydrogels

Dendrimer	*M*_w_^a^ (g/mol)	*M*_w_^b^ (g/mol)	HLB	log P^c^	Dendritic hydrogel	CD (%)^e^	SD (%)^f^
N_2_O_2_-G1V_4_ (**1**)	452.8	453.4	18.20	4.78	Hy[(N_2_O_2_-G1V_4_)x(P)] (**H1**)	80 (4 h)	300
N_2_O_2_-G2V_8_ (**2**)	901.8	901.6	6.80	13.66	Hy[(N_2_O_2_-G2V_8_)x(P)] (**H2**)	90 (3 h)	173
					Hy[(N_2_O_2_-G2V_6_)x(P)]V_2_ (**H2-V_**2**_**)	78 (5 h)	163
					Hy[(N_2_O_2_-G2V_4_)x(P)]V_4_ (**H2-V**_**4**_)	36 (4 h)	166
					Hy[(N_2_O_2_-G2V_6_)x(P)](NMe_2_HCl)_2_ (**H2-N**_**2**_)	-	200
					Hy[(N_2_O_2_-G2V_4_)x(P)](NMe_2_HCl)_4_ (**H2-N**_**4**_)	-	290
N_2_O_2_-G3V_16_ (**3**)	1800.0	1798.1	1.00	31.42	Hy[(N_2_O_2_-G3V_16_)x(P)] (**H3**)	85 (6 h)	180
Si-G0V_4_ (**4**)	136.3	d	d	3.42	Hy[(Si-G0V_4_)x(P)] (**H4**)	90 (1.5 h)	165
					Hy[(Si-G0V_3_)x(P)]V_1_ (**H4-V**_**1**_)	73 (2 h)	225
Si-G1V_8_ (**5**)	585.3	d	d	12.30	Hy[(Si-G1V_8_)x(P)] (**H5**)	90 (6 h)	149
Si-G2V_16_ (**6**)	1483.2	d	d	30.06	Hy[(Si-G2V_16_)x(P)] (**H6**)	40 (6 h)	201

a Calculated values from ChemDraw.

b Results from MALDI-TOF (M+H^+^).

c Predicted by ChemAxon.

d Not calculated.

e Average value from 6 different specimens.

f Average value from 2 different specimens.

#### 2.3.2 Hydrogel TEC Functionalization

2-(Dimethylamino)ethanethiol hydrochloride (2 eq. for H2-V_2_; 4 eq. for H2-V_4_) was added into a mixture of MeOH/THF/H_2_O (1/0.5/0.5). Next, DMPA (5 mol % of pendant vinyl groups) was added. The hydrogels were UV-irradiated for 4 and 6 h, respectively, and then extensively washed with MeOH until the DMPA was eliminated, as confirmed by TLC (hexane:ethyl acetate, 80:20).

#### 2.3.3 Hydrogel FPE Functionalization

##### 2.3.3.1 Ibuprofen Binding to **Hydrogel H2-V**_**2**_

Ibuprofen (7.5 mg, 1.5 equiv/OH gel) was previously activated by reaction with CDI (8.44 mg, 1.5 equiv/OH gel) in ethyl acetate, for 30 min at 50 °C, to generate the imidazolide derivative of ibuprofen. Subsequently, the CsF catalyst (0.74 mg, 0.2 equiv/OH gel) is added. The hydrogel (47.5 mg) was exposed to the solution for 20 h at 50 °C, with slight stirring (150 rpm). The hydrogel was purified by washing with water and acetone and dried under vacuum, obtaining a yellow hydrogel.

##### 2.3.3.2 Caffeic Acid Binding to Hydrogel **H2-V**_**2**_

Caffeic acid acetonide (5.76 mg, 1.5 equiv/OH gel) was previously activated by reaction with CDI (5.70 mg, 1.5 equiv/OH gel) in ethyl acetate, for 30 min at 50 °C, to generate the caffeic imidazolide derivative. Subsequently, the CsF catalyst (0.5 mg, 0.2 equiv/OH gel) was added. The hydrogel (32.0 mg) was exposed to the solution for 20 h at 50 °C, with slight stirring (150 rpm). The hydrogel was purified by washing with water and acetone and dried under a vacuum, obtaining a brown hydrogel that confirms the anchoring of caffeic acid.

### 2.4 Antibiotic Release Assays

#### 2.4.1 Enzyme-Promoted Ester Cleavage

The selected hydrogel (45.9 mg of **Ibu-H2-V**_**2**_; 29.6 mg of **Caf-H2-V**_**2**_) was immersed in 500 μL of water solution comprising 20% fetal bovine serum (FBS) and stirred under orbital shaking at 37 °C. Samples (50 μL) from the solution were taken over time. HPLC was then employed to study the release of ibuprofen or caffeic acid, as detailed in the Supporting Information.

#### 2.4.2 Ciprofloxacin Release

The selected hydrogel (**H2**) was immersed in 3 mL of water and stirred under orbital shaking at 37 °C. Samples (50 μL) from the solution were taken over time, and HPLC was employed to study the release.

### 2.5 Inhibition Zone Assay

A single colony of *S. aureus* from the agar plate was suspended into MHB II broth and incubated with shaking at 37 °C until the log phase concentration. The bacterial solution was then diluted with broth to reach a concentration of 10^6^ CFU mL^–1^. The bacteria solution was spread into the plates. Then, the different CIP-loaded hydrogels and the controls were placed on the plates and incubated at 37 °C for 18 h. Afterward, selected hydrogels were transferred onto the agar plate inoculated with bacteria. After incubation for 18 and 72 h, the diameters of the inhibition zones were measured, and the images were recorded in triplicate for each independent set.

## 3 Results and Discussion

### 3.1 Synthesis and Characterization of Bifunctional Carbosilane Dendrimers with a N_2_O_2_ Core

Carbosilane dendrimers present great kinetic stability, high flexibility, and very low polarity.^20^ For example, the traditional silicon-core dendrimers Si-G1V_8_ and Si-G2V_16_ present high partition coefficient (log *P*) values (12.30 and 30.06), revealing their high lipophilicity. In most cases, these dendrimers need to be functionalized with moieties that provide water-solubility, especially for biomedical applications. These functional groups are very often located at the periphery of the dendritic scaffold, taking advantage of the multivalence of the system. However, to our knowledge, no examples of carbosilane dendrimers presenting a core available for subsequent functionalization have been described in the literature.

**Scheme 1:**
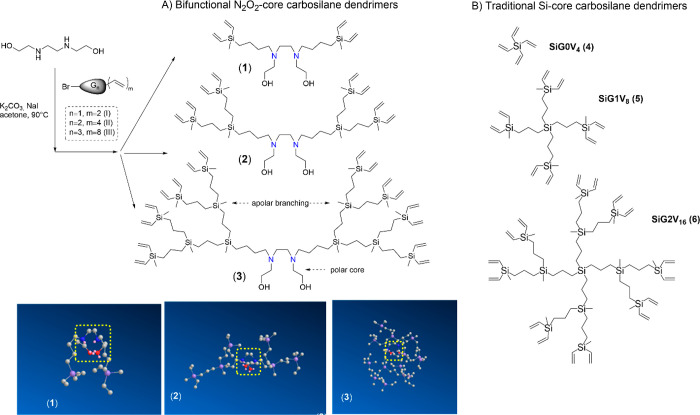
Carbosilane Dendrimers Used as Cross-Linkers in Dendritic Hydrogels: (A) Synthesis of Bifunctional Carbosilane Dendrimers N_2_O_2_-GnV_m_ (**1**–**3**) and (B) Traditional Si-Core Carbosilane Dendrimers Si-GnV_m_ (**4**–**6**) The insets show the snapshots from 3D spatial arrangement of each dendrimer after the molecular dynamics job. The dendrimer core is highlighted [Job 1 (Minimize Energy to Minimum RMS Gradient of 0.010) + Job 2 (Molecular Dynamics. Step Interval: 2.0 fs. Frame Interval: 10 fs. Terminate After: 10000 steps. Heating/Cooling Rate: 1.000 kcal/atom/ps. Target Temperature: 300 K)].

In order to expand the applications of carbosilane dendritic scaffolds, we herein designed a family of bifunctional dendrimers comprising the *N*,*N*′-bis(2-hydroxyethyl) ethylenediamine core (called N_2_O_2_ for simplicity). These dendrimers exhibit amphiphilic properties having a nonpolar zone (dendritic branches) and a polar region (the core) (Scheme 1A). Furthermore, they present two different types of functional groups (hydroxyls at the core and alkenes at the periphery), which can selectively react. In analogy to their dendron precursors, these dendrimers are called N_2_O_2_-G1V_4_ (**1**), N_2_O_2_-G2V_8_ (**2**), and N_2_O_2_-G3V_16_ (**3**).

The synthesis of dendrimers **1**–**3** was carried out through a convergent approach (Scheme 1A). The corresponding precursor dendron (BrG1V_2_ (**I**), BrG2V_4_ (**II**), or BrG3V_8_ (**III**), 2 mmol)^24^ and *N*,*N*′-bis(2-hydroxyethyl) ethylenediamine (1 mmol) were dissolved in the minimum amount of acetone. K_2_CO_3_ (3 mmol) and NaI (2 mmol) were added, and the reaction proceeded at 90 °C for 4–6 h. The reaction was monitored by ^1^H NMR, through the disappearance of the signal assigned to CH_2_–Br at 3.40 ppm and the appearance of CH_2_–N at 2.45 ppm. Once the reaction was finished, the solution was filtered and the solvent was evaporated. After purification through size-exclusion chromatography, the resultant dendrimers **1**–**3** were isolated as yellow oils with 75% yield. Dendrimers were characterized by ^1^H, ^13^C NMR, HSQC, elemental analysis, and mass spectrometry. Detailed protocols for the synthesis and characterization of all dendrimers appear in the Experimental Section and the Supporting Information (Figures S1–12).

As summarized in Table 1, in the amphiphilic dendrimers **1**–**3**, the hydrophilic–lipophilic balance (HLB) decreases with increasing generation, from the more hydrophilic G1 to the quite hydrophobic G3. As expected, the partition coefficient (log *P*) follows the inverse trend, from 4.78 of G1 (**1**) to 31.42 of G3 (**3**). Similar values were obtained for the Si-core family, from 3.42 of G0 (**4**) to 30.06 of G2 (**6**). Due to their multifunctional nature, N_2_O_2_-core dendrimers **1**–**3** exhibit relevant differences compared to Si-core dendrimers **4**–**6**. For example, the presence of two nitrogen atoms in the core provides acid–base properties to these molecules, with two differentiated p*K*_a_ values (5.14 and 9.33) as predicted by MarvinSketch 22.7 (Figures S4, S8, and S12). This indicates that in water solution at physiological pH 7.4, one of the two nitrogen atoms will be protonated. Furthermore, the 3D arrangement of the dendrimers is different, which will probably affect the cross-linking potential. For dendrimers **1**–**3**, the molecular dynamics (MD) simulations performed with Chem3D 22.0.0 confirm that the dendrimer core exhibits a “caging effect” (Scheme 1A, inset), while the carbosilane branches are oriented in different directions.

### 3.2 Synthesis of Multifunctional Dendritic Hydrogels

TEC is an outstanding tool for the efficient synthesis and decoration of dendrimers,^25^ polymers, and dendritic networks, with high efficiency and operability.^22,23^ In order to cross-link the vinyl-functional dendrimers into the desired networks, we employed the dithiol-functional poly(ethylene glycol) PEG1k(SH)_2_ (**7**). This polymer was synthesized through the esterification of PEG1k with 3-mercaptopropionic acid, in toluene at 110 °C.^26^ MALDI-TOF spectra confirmed the correct attachment of the −COO(CH_2_)_2_SH groups to the two terminal hydroxyls of the polymer (Figure S13). Importantly, the presence of ester bonds in the polymer provides degradable properties to the hydrogels.

**Figure 1:**
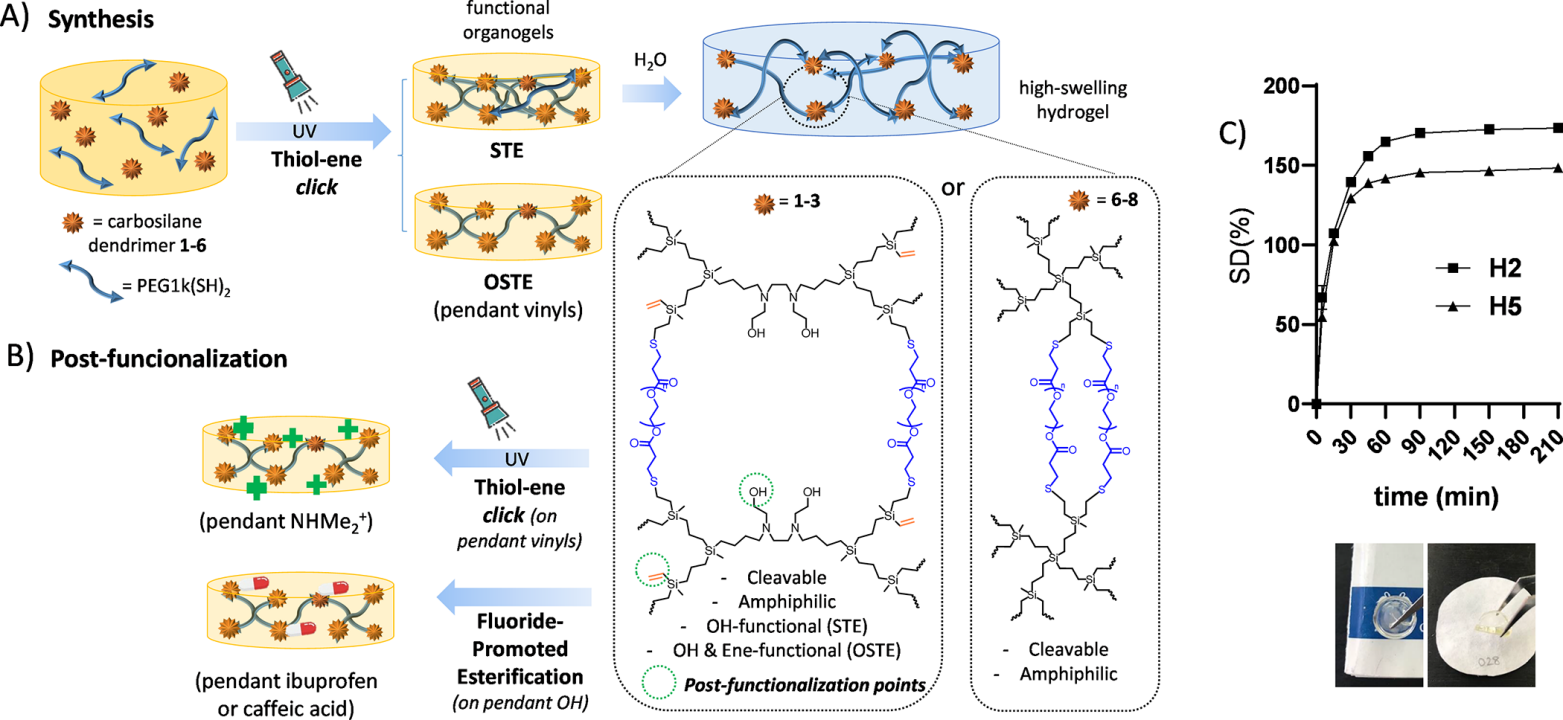
(A) Preparation of high-swelling dendritic hydrogels based on UV-initiated TEC, through STE or OSTE approaches. The nature (type, generation) of carbosilane dendrimer offers different postfunctionalization possibilities. (B) Postfunctionalization possibilities of the hydrogels. (C) Swelling degree over time in water at 25 °C for hydrogels H2 and H5 and image showing the aspect of H2.

The preparation of the hydrogels was carried out through UV-initiated TEC in a THF:MeOH (1:2) mixture and using DMPA as the photoinitiator, Figure 1A. A summary of the synthesized hydrogels and related properties is depicted in Table 1. A nomenclature for dendritic hydrogels is herein proposed, using the formula Hy[(D)x(P)], where *Hy* stands for “hydrogel”, *D* is the precursor dendrimer, and *P* is the polymer employed. In this work, the polymer was always PEG1k(SH)_2_, so it will be represented as P in the formula for simplicity. The dendritic moieties used for cross-linking appear within brackets while those available for functionalization are outside brackets. The efficiency of the reaction was represented by the cross-linking degree (CD%) while their ability to swell in water without dissolving was represented as the swelling degree (SD%).

For the preparation of the hydrogels, two different approaches were explored: Stoichiometric Thiol–Ene (STE) and nonstoichiometric Thiol–Ene (OSTE) conditions.

#### 3.2.1 STE Approach

In this route, we employed stoichiometric equivalents of thiol and vinyl moieties, aiming to achieve full conversion into thioether bonds. To evaluate the feasibility of the approach, Si-G1V_8_ (**5**, 1 mmol) and PEG1k(SH)_2_ (4 mmol) were dissolved in THF:MeOH. Then, DMPA (5 mol % alkenes) was added. The resulting mixture was added into several Teflon plugs with a capacity of 200 μL and exposed to the UV lamp until full cross-linking (6 h). Then, the hydrogels were washed several times with acetone until DMPA was fully eliminated and dried to obtain **H5** as a yellow network with CD 90% and SD 149% (Figure 1C).

A similar protocol was performed by using dendrimer N_2_O_2_-G2V_8_ (**2**). Hydrogel **H2** was formed in 3 h and isolated as a colorless material with CD 90% and SD 173% (Figure 1C). These examples serve to illustrate the impact of the dendritic scaffold, achieving a faster cross-linking of the 8 vinyl groups with N_2_O_2_-dendrimers. The more extended conformation and the amphiphilic nature, which favors the miscibility with PEG polymer, may be responsible for this faster reaction. Raman-confocal studies confirmed the disappearance of the −SH peak at 2582 cm^–1^ and the almost complete removal of the −CH=CH_2_ peak at 1596 cm^–1^ (Figure S14). In the STE approach, we accomplished networks with pendant hydroxyl groups from the dendritic cores, while all alkene groups were employed for cross-linking the network. However, the dendrimers also enable a precise control on the peripheral vinyl groups, which could be used for cross-linking but also for postfunctionalization. The OSTE approach delivers dual-functional networks bearing both hydroxyl- and vinyl-pendant moieties, as described below.

#### 3.2.2 OSTE Approach

In this approach, we used a controlled excess of alkene groups to generate vinyl-functional networks, which could be functionalized in subsequent steps (Figure 1B). Using a protocol similar to the STE approach, dendrimer N_2_O_2_-G2V_8_ (**2**) was reacted with 2 or 3 mmol of PEG1k(SH)_2_, to generate hydrogels **H2-V**_**4**_ and **H2-V**_**2**_, respectively. From the available 8 vinyl groups in each dendrimer, 4 or 6 were employed for cross-linking, while 4 or 2 were kept unreacted. Unlike the STE counterpart **H2**, OSTE hydrogels are yellowish materials due to the unreacted alkenes, with a less solid appearance even after longer UV-exposure (4 h). Furthermore, the CD% decreases with the number of cross-linked vinyls: 90% in **H2** to 78% in **H2-V**_**2**_ and 36% in **H2-V**_**4**_. In this case, Raman spectra revealed the presence of the −CH=CH_2_ peak at 1596 cm^–1^, more intense in **H2-V**_**4**_ according to the presence of more unreacted vinyls (Figure S15).

### 3.3 Postfunctionalization of Dendritic Hydrogels

The dendritic hydrogels herein designed offer multiple opportunities for functionalizing the networks with interesting moieties to provide antibacterial action. To confirm the viability of the approach, selected molecules were attached to the pendant alkenes or hydroxyl groups (Figure 1B), as described below.

#### 3.3.1 Thiol–Ene on Pendant Alkenes

To test the feasibility of this approach, 2-(dimethylamino)ethanethiol chloride (1.2 mol/mol alkene) and DMPA (5 mol %/mol alkene) were dissolved in a mixture MeOH:THF:H_2_O (1/0.5/0.5). Next, the OSTE hydrogels **H2-V**_**2**_ and **H2-V**_**4**_ were immersed in this mixture and exposed to UV irradiation for 4 h. Then, the hydrogels were washed with MeOH until complete removal of DMPA and other byproducts. The resultant hydrogels, **H2-N**_**2**_ and **H2-N**_**4**_, present pendant NHMe_2_^+^ groups with potential antibacterial activity. Unfortunately, the intrinsic fluorescence from the new ammonium groups hindered accurate characterization through Raman spectroscopy.

#### 3.3.2 Esterification on Pendant Hydroxyls

We have recently described the advantages of using fluoride-promoted esterification (FPE) to attach a drug to dendritic hydrogels.^18^ FPE is a versatile, robust, and clean approach, which is herein used to esterify ibuprofen (Ibu) and caffeic acid (Caf) as model compounds with available −COOH groups. Ibuprofen is a traditional NSAID drug which also has a relevant antimicrobial activity^27^ while caffeic acid is a natural antioxidant with potent antimicrobial effect.^28^

Ibuprofen (1.5 mol/mol OH gel, 7.5 mg) was activated by reaction with CDI (1.5 mol/mol OH gel) in ethyl acetate, for 30 min at 50 °C, to generate the imidazolyde derivative. Subsequently, CsF catalyst was added (0.2 mol/mol of OH gel). Hydrogel **H2-V**_**2**_ (47.5 mg) was immersed in the solution and kept at 50 °C, with slight stirring (150 rpm) for 20 h. The hydrogel was removed, washed with water and acetone, and then vacuum-dried, obtaining a yellow material. The hydrogel Hy[(N_2_(OIbu)_2_-G2V_6_)x(P)]V_2_ (**Ibu-H2-V**_**2**_) was obtained. A similar approach was employed to attach acetonide-protected caffeic acid, leading to brown hydrogel Hy[(N_2_(OCaf)_2_-G2V_6_)x(P)]V_2_ (**Caf-H2-V**_**2**_). FT-IR spectra confirmed the success of the esterification step, appearing an intense band around 1750 cm^–1^ assigned to the ester bonds, and the disappearance of the broad band around 3300 cm^–1^ from the free hydroxyl groups (Figures S16 and S17).

**Figure 2:**
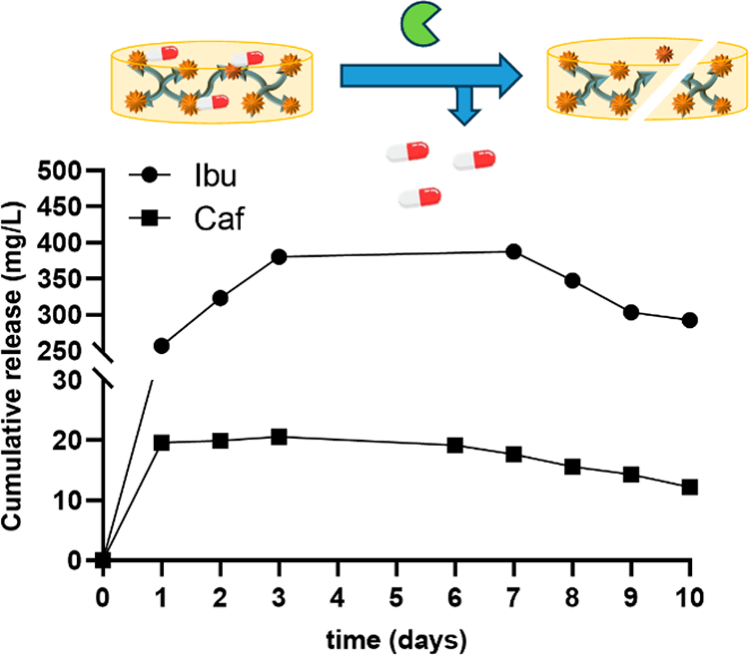
Esterase-mediated release of ibuprofen and caffeic acid from the OSTE hydrogel **H2-V**_**2**_.

The drug release from the esterified hydrogels was then explored. Hydrogels **Ibu-H2-V**_**2**_ and **Caf-H2-V**_**2**_ were immersed in water solution comprising 20% fetal bovine serum (FBS) and stirred under orbital shaking at 37 °C. Samples (50 μL) from the solution were taken over time, and HPLC was then employed to quantify drug release (Figure 2). Relevant differences were observed between these two molecules. The maximum released concentration is 20.6 mg/L for caffeic acid, while for ibuprofen it is 380 mg/L. Furthermore, the stronger release was found in the first 24 h for caffeic acid, while 72 h for ibuprofen was required to reach equilibrium. FT-IR also confirmed the release of the compounds, with a decrease of the band around 1750 cm^–1^, and the appearance of the band around 3300 cm^–1^ from the free hydroxyl groups (Figures S16 and S17). These results support the potential of OSTE hydrogels as carriers of covalently bound molecules with antimicrobial activity.

### 3.4 Swelling Degree under Different Conditions: Impact of Structure, pH, and Temperature

To further evaluate the properties and potential uses of the dendritic hydrogels, we explored the SD% under different conditions. The results herein summarized confirmed the impact of (A) the dendrimer generation; (B) the nature and number of pendant groups; (C) the pH; and (D) the temperature. A full discussion is included in the Supporting Information (Figure S19).

For STE hydrogels **H1**–**H3**, the SD values decreased when increasing the dendrimer generation and the corresponding lipophilicity, from 300% in **H1** to 180% in **H3** (Figure S19A). **H2** and **H3** exhibited a similar behavior, due to the high lipophilicity of both dendrimers **2** and **3** that prevent a comfortable loading of water within the pores of the hydrogels. For OSTE hydrogels (Figure S19B), the vinyl-functional hydrogels **H2-V**_**2**_ and **H2-V**_**4**_ exhibited the same swelling than the STE counterpart **H2**, around 150%. However, when functionalized with ammonium groups, the SD increased to 200% for **H2-N**_**2**_ and 290% for **H2-N**_**4**_. The electrostatic repulsion produced by close cationic groups favors the increase in swelling degree. Additionally, the presence of nitrogen atoms within the network can produce a pH-responsive behavior. The SD% of selected hydrogels (**H2-V**_**2**_ and **H2-N**_**2**_) was studied in two different buffer solutions at pH 5.5 and 7.4, compared with unbuffered water (Figure S19C). Subtle changes occur for **H2-V**_**2**_ under the three different conditions, while a more pronounced effect is observed for **H2-N**_**2**_, with pendant ammonium groups. A 30% higher swelling is found at pH 5.5 compared to pH 7.4, due to the repulsion of the cationic groups. The total protonation of the nitrogen atoms produces an expansion of the dendritic core. For example, in dendrimer **1**, the N–N distance grows from 2.851 to 3.906 Å after protonation, as well as the O–O distance from 5.559 to 9.827 Å (Figure S18). This pH-responsive behavior can be useful in drug encapsulation and drug release studies as well as the temperature-responsive properties. At 37 °C, all hydrogels exhibited a significantly lower swelling than at 25 °C (Figure S19D). This effect is especially relevant for ammonium-functional hydrogels **H2-N**_**2**_ and **H2-N**_**4**_, which underwent a 45% and 77% reduction of swelling, respectively.

### 3.5 Antibiotic Encapsulation and Release Studies

A broad list of common antibiotics presents poor solubility in water. This clearly affects the bioavailability, the frequency of dosing, as well as the patient compliance. Having a hydrogel reservoir of the antibiotic, which can sustainably release the drug, may reduce the side effects, increase the efficacy of the drug, and thus contribute to a decreased AMR. The amphiphilic nature of these hydrogels may also improve the compatibility with low polarity drugs, compared with traditional hydrophilic hydrogels. Herein, we explored the ability of the dendritic hydrogels to encapsulate ciprofloxacin (CIP) as model antibiotic. CIP is a fluoroquinolone with broad-spectrum activity but is barely soluble in water (<1 mg/mL);^29^ thus it is usually administered in the hydrochloride form to increase its solubility.

#### 3.5.1 Antibiotic Loading

Considering the poor solubility of CIP in most solvents, we maximized the loading in the hydrogels by performing the encapsulation in warm chloroform, as the optimal conditions.^30^ The selected hydrogel was immersed in a solution of CIP in chloroform (1.1 mg in 3 mL) for 60 h at 40 °C, and subsequently, the solvent was evaporated. HPLC studies confirmed between 52 and 95% loaded CIP.

#### 3.5.2 Antibiotic Release Studies

**Figure 3:**
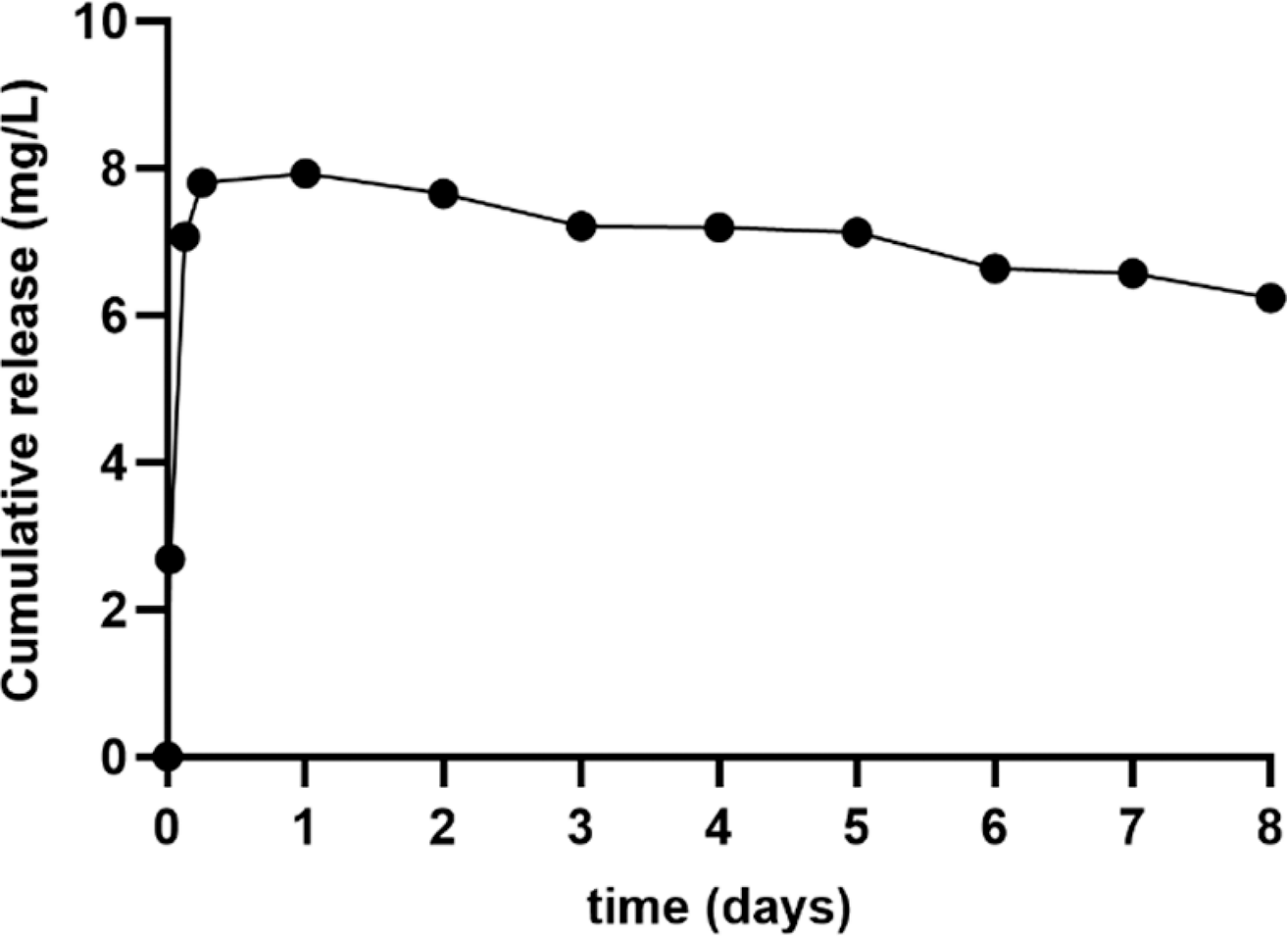
Ciprofloxacin release in solution.

First, CIP-encapsulated hydrogels were immersed in a water solution (3 mL), and small aliquots (50 μL) were collected at different times. After every collection, 50 μL of water was added to keep the volume constant. Then the CIP concentration was quantified through HPLC. As displayed in Figure 3, CIP exhibited a burst release in the first 6 h and a sustained release until day 8. Compared to covalently bound drugs, encapsulation produces a faster release but a lower loading of the hydrogel.

### 3.6 Evaluation of the Antibacterial Activity of Dendritic Hydrogels

**Figure 4:**
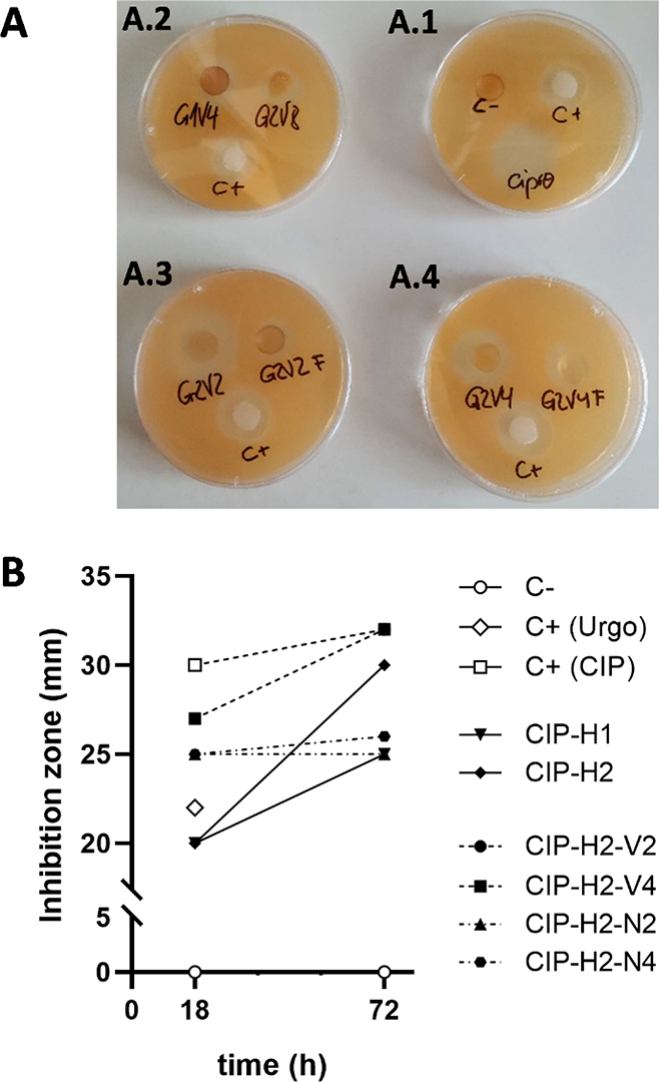
(A) *S. aureus* plates showing the inhibition halos due to the ciprofloxacin release during 18 h from different hydrogels. (A.1) Controls: C– (unloaded **H2-N**_**2**_), C+ (Urgo patch), and ciprofloxacin (1 mg/mL). (A.2) STE hydrogels CIP-**H1** and CIP-**H2**. (A.3) OSTE hydrogels CIP-**H2-V**_**2**_ and CIP-**H2-N**_**2**_. (A.4) OSTE hydrogels CIP-**H2-V**_**4**_ and CIP-**H2-N**_**4**_. (B) Comparison of inhibition zones at 18 and 72 h.

The antibacterial activity of selected hydrogels was evaluated against *Staphylococcus aureus* (Gram+ bacteria) using an inhibition zone assay (see Experimental Section). As a negative control, hydrogel **H2-V**_**2**_ (C−) was used. As positive controls, both ciprofloxacin (1 mg/mL) and the commercial dressing Urgo Resistant Antiseptic (C+, 1 cm diameter) were employed. The commercial patch contains the antibiotic benzalkonium chloride at 960 mg/m^2^. Then, different CIP-loaded hydrogels were tested at 18 and 72 h. For the drug encapsulation, a saturated CIP solution in chloroform was prepared (0.78 mg/mL at 50 °C)^30^ and the hydrogel (65 mg) was immersed in 1 mL of the solution for 20 h at 50 °C with slight stirring (150 rpm). Subsequently, the hydrogel was dried under vacuum and the amount of drug retained was quantified, with values of 1 mg CIP/100 mg hydrogel. The results are depicted in Figure 4.

After 18 h, STE hydrogels **H1** and **H2** (Figure 4A.2) showed inhibition zones similar to those of the positive control patch, confirming the diffusion of the antibiotic to the medium. The OSTE hydrogels **H2-V**_**2**_ and **H2-V**_**4**_ (Figures 4A.3,4) showed higher inhibition zones than the commercial control, reaching values close to those of the ciprofloxacin directly applied to the plate. Finally, the ammonium-functionalized counterparts **H2-N**_**2**_ and **H2-N**_**4**_ (Figures 4A.3,4) exhibited a smaller inhibition zone but again close to the commercial control. The explanation can be found in the strong electrostatic interactions established between the cationic −NHMe_2_^+^ pending groups in the hydrogels and the carboxylic acid in the drug.

After 72 h, we observed a sustained release of CIP for most hydrogels (Figure 4B). The release is especially relevant for STE hydrogels **H1** and **H2**, where the inhibition zone increased 5 and 10 mm, respectively. For **H2-V**_**2**_ and **H2-V**_**4**_, a sustained release is also observed (increase of 2 and 5 mm, respectively), while for the ammonium functionalized hydrogels **H2-N**_**2**_ and **H2-N**_**4**_ are less pronounced due to the strong drug–hydrogel interactions. All of these results confirm the impact of the hydrogel structure and the pendant groups on the drug release.

**Figure 5:**
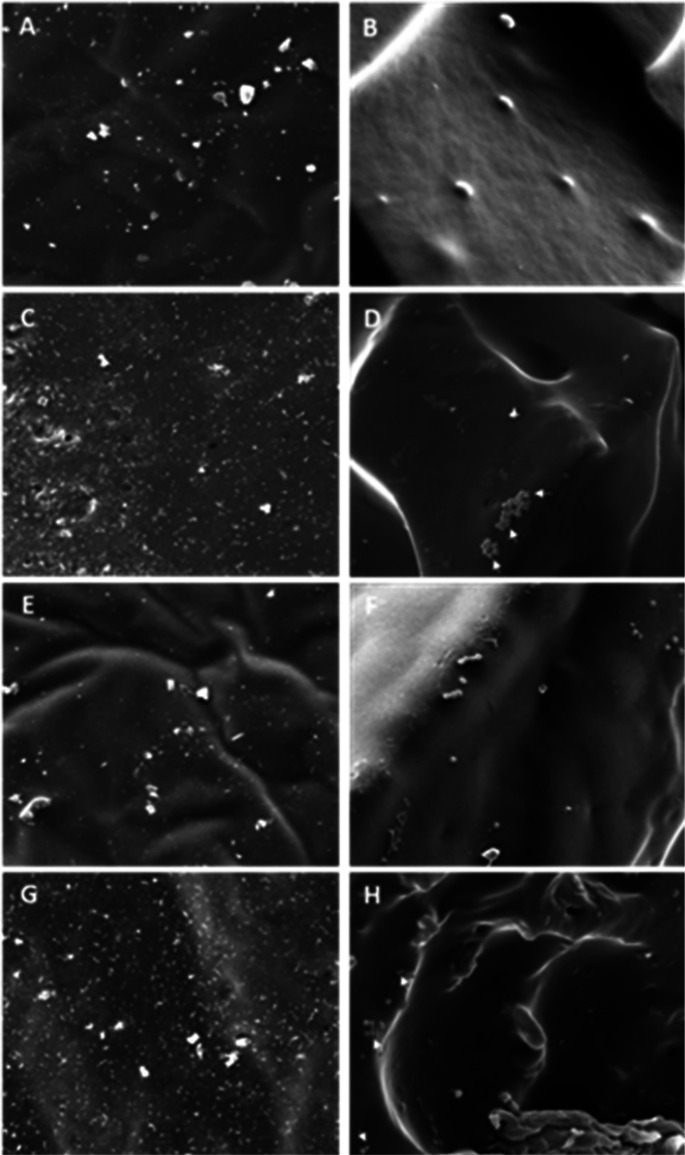
SEM images of **H2-V**_**2**_ and **CIP-H2-V**_**2**_ after a 24 h inhibition zone assay. **H2-V**_**2**_ top (A), top cross-section (B), bottom (E), and bottom cross-section (F). **CIP-H2-V**_**2**_ top (C), top cross-section (D), bottom (G), bottom cross-section (H). (Arrows: cell morphology altered.)

Subsequently, we selected a pristine hydrogel (**H2-V**_**2**_) and its loaded counterpart (**CIP-H2-V**_**2**_) and performed scanning electron microscopy (SEM) studies to explore the presence of bacterial cells within the networks (Figure 5). On the one hand, top and bottom **H2-V**_**2**_ (Figure 5A,E) and **CIP-H2-V**_**2**_ (Figure 5C,G) images did not show the presence of bacteria as expected. In the case of bottom samples, the bacteria that were spread on the agar surface could not grow in these conditions and may be absorbed by the gel structure during the swelling process. On the other hand, **H2-V**_**2**_ (Figure 5B,F) and **CIP-H2-V**_**2**_ (Figure 5D,H) perpendicular cross sections showed the presence of bacteria, suggesting cell penetration within the hydrogel. These cells exhibited a normal shape in negative control hydrogels (Figure 5B,F). However, the morphology of those located inside the ciprofloxacin-loaded hydrogel was altered, as some collapsed and smaller bacteria were observed (Figure 5D,H; see arrows).

The advantages of formulating ciprofloxacin as a hydrogel have been previously demonstrated in the literature. For example, a CIP-based hyperbranched polymer hydrogel exhibited greater efficacy in the solid state than in the liquid state, due to the forced contact between the cell wall and the gel surface.^31^ However, although this hydrogel showed a potent effect against *Vibrio chemaguriensis*, no activity was found against *E. coli* and *S. aureus*. Another example is the photo-cross-linkable gelatin-based hydrogel GelCORE, used for ocular applications. GelCORE was loaded with CIP-containing micelles and exhibited a drug release for up to 24 h.^32^ It showed excellent antibacterial properties against *S. aureus* with inhibition zones of 35.5 mm. The results found with our dendritic hydrogels reveal a longer sustained release of CIP which can be fine-tuned through the nature and number of pendant groups in the network.

## 4 Conclusions

The new bifunctional carbosilane dendrimers presented herein are useful tools for the preparation of dendritic hydrogels. They enable the precise design of the networks, which exhibit on-demand moieties that provide temperature- and pH-responsive properties. This versatility can be exploited to generate antibacterial materials from different angles. Herein, we showed that they can encapsulate ciprofloxacin and slowly release it, generating an inhibition zone against *Staphylococcus aureus*. These amphiphilic hydrogels could open new avenues in the topical treatment of infections using antibiotics with poor solubility in water. Additionally, these hydrogels can also covalently attach different compounds and subsequently release them under the presence of esterases. This enables a higher loading as well as a higher control of the drug release, expanding even further their potential uses. Overall, carbosilane-based dendritic hydrogels appear as a promising strategy to address the global health issue of antibiotic resistance.

## Supporting Information Available

Detailed materials and methods; ^1^H, ^13^C, and HSQC NMR spectra of dendrimers **1**–**3**; and characterization of hydrogels (swelling degree, drug release curves, FTIR spectra) (PDF)

## References

[ref1] Gaynes R. The Discovery of Penicillin—New Insights After More Than 75 Years of Clinical Use. Emerg. Infect. Dis. 2017, 23, 849–853. 10.3201/eid2305.161556.

[ref2] Murray C. J. L.; et al. Global burden of bacterial antimicrobial resistance in 2019: a systematic analysis. Lancet 2022, 399, 629–655. 10.1016/S0140-6736(21)02724-0. 35065702 PMC8841637

[ref3] Pancu D. F.; Scurtu A.; Macasoi I. G.; Marti D.; Mioc M.; Soica C.; Coricovac D.; Horhat D.; Poenaru M.; Dehelean C. Antibiotics: Conventional Therapy and Natural Compounds with Antibacterial Activity-A Pharmaco-Toxicological Screening. Antibiotics 2021, 10, 401 10.3390/antibiotics10040401. 33917092 PMC8067816

[ref4] Ghosh C.; Sarkar P.; Issa R.; Haldar J. Alternatives to Conventional Antibiotics in the Era of Antimicrobial Resistance. Trends Microbiol. 2019, 27, 323–338. 10.1016/j.tim.2018.12.010. 30683453

[ref5] Godoy-Gallardo M.; Eckhard U.; Delgado L. M.; de Roo Puente Y. J. D.; Hoyos-Nogués M.; Gil F. J.; Perez R. A. Antibacterial approaches in tissue engineering using metal ions and nanoparticles: From mechanisms to applications. Bioact. Mater. 2021, 6, 4470–4490. 10.1016/j.bioactmat.2021.04.033. 34027235 PMC8131399

[ref6] Song J.; Jang J. Antimicrobial polymer nanostructures: synthetic route, mechanism of action and perspective. Adv. Colloid Interface Sci. 2014, 203, 37–50. 10.1016/j.cis.2013.11.007. 24332622

[ref7] Gan B. H.; Gaynord J.; Rowe S. M.; Deingruber T.; Spring D. R. The multifaceted nature of antimicrobial peptides: current synthetic chemistry approaches and future directions. Chem. Soc. Rev. 2021, 50, 7820–7880. 10.1039/D0CS00729C. 34042120 PMC8689412

[ref8] Li S.; Dong S.; Xu W.; Tu S.; Yan L.; Zhao C.; Ding J.; Chen X. Antibacterial hydrogels. Adv. Sci. 2018, 5 (5), 1700527 10.1002/advs.201700527. PMC5980143 29876202

[ref9] Liu J.; Jiang W.; Xu Q.; Zheng Y. Antibacterial Hydrogels. Gels 2022, 8, 503 10.3390/gels8080503. 36005104 PMC9407327

[ref10] Caló E.; Khutoryanskiy V. V. Biomedical applications of hydrogels: A review of patents and commercial products. Eur. Polym. J. 2015, 65, 252–267. 10.1016/j.eurpolymj.2014.11.024.

[ref11] Larrañeta E.; Stewart S.; Ervine M.; Al-Kasasbeh R.; Donnelly R. F. Hydrogels for Hydrophobic Drug Delivery. Classification, Synthesis and Applications. J. Funct. Biomater. 2018, 9, 13 10.3390/jfb9010013. 29364833 PMC5872099

[ref12] Kaga S.; Arslan M.; Sanyal R.; Sanyal A. Dendrimers and Dendrons as Versatile Building Blocks for the Fabrication of Functional Hydrogels. Molecules 2016, 21, 497 10.3390/molecules21040497. 27092481 PMC6273238

[ref13] Zhang Y. S.; Khademhosseini A. Advances in engineering hydrogels. Science 2017, 356, eaaf3627 10.1126/science.aaf3627. 28473537 PMC5841082

[ref14] Ghobril C.; Rodriguez E. K.; Nazarian A.; Grinstaff M. W. Recent Advances in Dendritic Macromonomers for Hydrogel Formation and Their Medical Applications. Biomacromolecules 2016, 17, 1235–1252. 10.1021/acs.biomac.6b00004. 26978246

[ref15] Malkoch M.; García-Gallego S. Dendrimer Chemistry: Synthetic Approaches Towards Complex Architectures; The Royal Society of Chemistry: London, U.K., 2020; Vol. 1, p 293.

[ref16] Navath R. S.; Menjoge A. R.; Dai H.; Romero R.; Kannan S.; Kannan R. M. Injectable PAMAM dendrimer-PEG hydrogels for the treatment of genital infections: formulation and in vitro and in vivo evaluation. Mol. Pharmaceutics 2011, 8, 1209–1223. 10.1021/mp200027z. PMC3556449 21615144

[ref17] McMahon S.; Kennedy R.; Duffy P.; Vasquez J. M.; Wall J. G.; Tai H.; Wang W. Poly(ethylene glycol)-Based Hyperbranched Polymer from RAFT and Its Application as a Silver-Sulfadiazine-Loaded Antibacterial Hydrogel in Wound Care. ACS Appl. Mater. Interfaces 2016, 8, 26648–26656. 10.1021/acsami.6b11371. 27636330

[ref18] Andrén O. C. J.; Ingverud T.; Hult D.; Håkansson J.; Bogestål Y.; Caous J. S.; Blom K.; Zhang Y.; Andersson T.; Pedersen E.; Björn C.; Löwenhielm P.; Malkoch M. Antibiotic-Free Cationic Dendritic Hydrogels as Surgical-Site-Infection-Inhibiting Coatings. Adv. Healthc. Mater. 2019, 8, 1801619 10.1002/adhm.201801619. 30735288

[ref19] Recio-Ruiz J.; Carloni R.; Ranganathan S.; Muñoz-Moreno L.; Carmena M. J.; Ottaviani M. F.; de la Mata F. J.; García-Gallego S. Amphiphilic Dendritic Hydrogels with Carbosilane Nanodomains: Preparation and Characterization as Drug Delivery Systems. Chem. Mater. 2023, 35, 2797–2807. 10.1021/acs.chemmater.2c03436. 37063594 PMC10101558

[ref20] de la Mata F. J.; Gómez R.; Cano J.; Sánchez-Nieves J.; Ortega P.; García-Gallego S. Carbosilane dendritic nanostructures, highly versatile platforms for pharmaceutical applications. WIREs Nanomed. Nanobiotechnol. 2023, 15, e1871 10.1002/wnan.1871. 36417901

[ref21] Ortega P.; Sánchez-Nieves J.; Cano J.; Gómez R.; de la Mata F. J. In Dendrimer Chemistry: Synthetic Approaches Towards Complex Architectures; Malkoch M., García-Gallego S., Eds.; The Royal Society of Chemistry: London, U.K., 2020; Chapter 5, pp 114–145.

[ref22] Mongkhontreerat S.; Öberg K.; Erixon L.; Löwenhielm P.; Hult A.; Malkoch M. UV initiated thiol–ene chemistry: a facile and modular synthetic methodology for the construction of functional 3D networks with tunable properties. J. Mater. Chem. A 2013, 1, 13732–13737. 10.1039/c3ta12963b.

[ref23] Granskog V.; García-Gallego S.; von Kieseritzky J.; Rosendahl J.; Stenlund P.; Zhang Y.; Petronis S.; Lyvén B.; Arner M.; Håkansson J.; Malkoch M. High-Performance Thiol–Ene Composites Unveil a New Era of Adhesives Suited for Bone Repair. Adv. Funct. Mater. 2018, 28, 1800372 10.1002/adfm.201800372.

[ref24] Fuentes-Paniagua E.; Peña-González C. E.; Galán M.; Gómez R.; de la Mata F. J.; Sánchez-Nieves J. Thiol-Ene Synthesis of Cationic Carbosilane Dendrons: a New Family of Synthons. Organometallics 2013, 32, 1789–1796. 10.1021/om301217g.

[ref25] García-Gallego S.; Andrén O. C. J.; Malkoch M. Accelerated Chemoselective Reactions to Sequence-Controlled Heterolayered Dendrimers. J. Am. Chem. Soc. 2020, 142, 1501–1509. 10.1021/jacs.9b11726. 31895981

[ref26] Macdougall L. J.; Pérez-Madrigal M. M.; Arno M. C.; Dove A. P. Nonswelling Thiol-Yne Cross-Linked Hydrogel Materials as Cytocompatible Soft Tissue Scaffolds. Biomacromolecules 2018, 19, 1378–1388. 10.1021/acs.biomac.7b01204. 29125285 PMC5954353

[ref27] Obad J.; Šušković J.; Kos B. Antimicrobial activity of ibuprofen: new perspectives on an ″Old″ non-antibiotic drug. Eur. J. pharm. Sci. 2015, 71, 93–98. 10.1016/j.ejps.2015.02.011. 25708941

[ref28] Khan F.; Bamunuarachchi N. I.; Tabassum N.; Kim Y. M. Caffeic Acid and Its Derivatives: Antimicrobial Drugs toward Microbial Pathogens. J. Agric. Food Chem. 2021, 69, 2979–3004. 10.1021/acs.jafc.0c07579. 33656341

[ref29] Caço A. I.; Varanda F.; Pratas de Melo M. J.; Dias A. M. A.; Dohrn R.; Marrucho I. M. Solubility of antibiotics in different solvents. Part II. non-hydrochloride forms of tetracycline and ciprofloxacin. Ind. Eng. Chem. Res. 2008, 47, 8083–8089. 10.1021/ie8003495.

[ref30] Zhang C.-L.; Zhao F.; Wang Y. Thermodynamics of the solubility of ciprofloxacin in methanol, ethanol, 1-propanol, acetone, and chloroform from 293.15 to 333.15 K. J. Mol. Liq. 2010, 156, 191–193. 10.1016/j.molliq.2010.06.004.

[ref31] Mukherjee I.; Ghosh A.; Bhadury P.; De P. Matrix-Assisted Regulation of Antimicrobial Properties: Mechanistic Elucidation with Ciprofloxacin-Based Polymeric Hydrogel Against Vibrio Species. Bioconjugate Chem. 2019, 30 (1), 218–230. 10.1021/acs.bioconjchem.8b00846. 30516978

[ref32] Khalil I. A.; Saleh B.; Ibrahim D. M.; Jumelle C.; Yung A.; Dana R.; Annabi N. Ciprofloxacin-loaded bioadhesive hydrogels for ocular applications. Biomater. Sci. 2020, 8, 5196–5209. 10.1039/D0BM00935K. 32840522 PMC7594650

