# Supplementary material for: Bifunctional Carbosilane Dendrimers for the Design of Multipurpose Hydrogels with Antibacterial Action
Source: Chem Mater. 2023 Dec 26;36(1):266–74. doi: 10.1021/acs.chemmater.3c02027 (PMC10783294; doi:10.1021/acs.chemmater.3c02027)
Supplement: Supplementary file 1 — cm3c02027_si_001.pdf [file cm3c02027_si_001.pdf]

Electronic Supporting Information

Bifunctional carbosilane dendrimers for the design of multipurpose hydrogels with antibacterial action

Silvia Muñoz-Sánchez,[a] *Irene Heredero-Bermejo,*[b] *Francisco Javier de la Mata* [a,c,d] *and Sandra García-Gallego** [a,c,d]

a. University of Alcala, Department of Organic and Inorganic Chemistry and Research Institute in Chemistry "Andrés M. Del Río" (IQAR), 28805, Madrid, Spain. silvia.munoz@uah.es

b. University of Alcala, Department of Biomedicine and Biotechnology, 28805, Madrid, Spain.

c. Networking Research Center on Bioengineering, Biomaterials and Nanomedicine (CIBER-BBN), 28029, Madrid, Spain;

d. Institute Ramón y Cajal for Health Research (IRYCIS), 28034, Madrid, Spain.

Materials and methods………………………………………………………………………………………………3

Figures………5

Figure S1. ^{1}H and ^{13}C NMR spectra of dendrimer N_2O_2-G1V_4 **(1)**.

Figure S2. ^{1}H-^{13}C HSQC spectra of dendrimer N_2O_2-G1V_4 **(1)**.

Figure S3. MALDI spectrum of dendrimer N_2O_2-G1V_4 **(1)**.

Figure S4. Prediction of pK_a values and microspecies distribution for dendrimer **1**.

Figure S5. ^{1}H and ^{13}C NMR spectra of dendrimer N_2O_2-G2V_8 **(2)**.

Figure S6. ^{1}H-^{13}C HSQC spectra of dendrimer N_2O_2-G2V_8 **(2)**.

Figure S7. MALDI spectra of dendrimer N_2O_2-G2V_8 **(2)**.

Figure S8. Prediction of pK_a values and microspecies distribution for dendrimer **2**.

Figure S9. ^{1}H and ^{13}C NMR spectra of dendrimer N_2O_2-G3V_{16} **(3)**.

Figure S10. ^{1}H-^{13}C HSQC spectra of dendrimer N_2O_2-G3V_{16} **(3)**.

Figure S11. MALDI spectra of dendrimer N_2O_2-G3V_{16} **(3)**.

Figure S12. Prediction of pKa values and microspecies distribution for dendrimer 3.

Figure S13. MALDI spectra of PEG1k$(SH)_2$ (**7**).

Figure S14. RAMAN-confocal spectra of polymeric precursor PEG1k$(SH)_2$ (**7**, A) and STE hydrogels **H4** (B), **H1** (C) and **H2** (D).

Figure S15. RAMAN-confocal spectra of OSTE hydrogels **H2-V_2** and **H2-V_4**.

Figure S16. FT-IR spectra of Hy[(N_2(OIbu)$_2$-G2V_6)x(P)]V_2 (**Ibu-H2-V_2**) before (top) and after (bottom) exposure to FBS-containing solution.

Figure S17. FT-IR spectra of Hy[(N_2(OCaf)$_2$-G2V_6)x(P)]V_2 (**Caf-H2-V_2**) before (top) and after (bottom) exposure to FBS-containing solution.

Figure S18. Snapshots from 3D spatial arrangement of: **A)** Dendrimer **1** with the two nitrogen atoms deprotonated (left) or protonated (right). **B)** Dendrimer **2** with the two nitrogen atoms deprotonated (left) or protonated (right). **C)** Dendrimer **3** with the two nitrogen atoms deprotonated (left) or protonated (right).

Figure S19. Swelling degree assays, to evaluate the impact of (A) the dendrimer generation; (B) the pendant groups; (C) the pH; and (D) the temperature.

Materials and methods.

Materials. Reagents and solvents were purchased from commercial sources and used as received. Poly(ethylene glycol) 1kD (PEG1k), *N,N'*-Bis(2-hydroxyethyl)ethylenediamine), 3-mercaptopropionic acid, *p*-toluenesulfonic acid monohydrate, potassium carbonate and ibuprofen were purchased from Sigma Aldrich. Sodium iodide (>99%) was purchased from Fisher Chemical. 2,2-dimethoxy-2-phenylacetophenone (DMPA) was purchased from Acros Organics. Toluene was purchased from PanReac. Chloroform was purchased from J.T.Baker. Acetone, tetrahydrofuran, and methanol were purchased from Sigma Aldrich with HPLC grade. Vinyl-decorated dendrons N_3GnV_m (**I-III**) were synthesized as previously reported.[1]

NMR spectroscopy. NMR spectra were acquired at CAIQ-UAH, in Varian NMR System-500, Varian Mercury Plus-300 and Bruker AVANCE Neo 400 instruments at room temperature and using $CDCl_3$ as solvent. The chemical shifts are expressed in ppm using the solvent as internal reference, in ^1H NMR ($CDCl_3$ δ (H)=7.24 ppm) and in ^{13}C NMR ($CDCl_3$ δ (C) = 77.0 ppm). HSQC were also carried out under these conditions.

Elemental analysis. Quantitative analyses of C, H and N were performed employing a LECO CHNS-932 microanalyzer.

[1] E. Fuentes-Paniagua, C. E. Peña-González, M. Galán, R. Gómez, F. J. de la Mata and J. Sánchez-Nieves, *Organometallics*, **2013**, 32(6), 1789-1796.

MALDI-TOF. Mass spectrometry assays were performed using a Bruker ULTRAFLEX III TOF/TOF. For samples preparation, the compounds were dissolved in acetone and then mixed with DCTB matrix and NaI.

RAMAN-confocal microscopy. Raman spectra were recorded using a Thermo Scientific DXR Raman confocal microscope, controlled by Thermo Scientific OMNIC 8.3.103 software for dispersive Raman, kindly provided by CINQUIFOR-UAH research group. A laser emitting at 780 nm with 10 mW and a confocal slot size of 50 μm was used. The microscope was set at 10 × magnification under bright field illumination. Raman spectra were recorded in the range 3300-400 cm^{-1} and subsequently normalized between 0 and 1 to facilitate comparison.

High Performance Liquid Chromatography (HPLC). Drug release studies were performed at CAIQ-UAH on an Agilent 1200 HPLC equipment. For ciprofloxacin, using an ACE Excel 5 column and a mobile phase of 0.2% trifluoroacetic acid and acetonitrile (30:70), with an injection volume of 5 μL. The drug is detected at a wavelength of 280 nm. For ibuprofen, using the same column and mobile phase acetonitrile and phosphoric acid (70:30), with an injection volume of 10 μL. The drug is detected at a wavelength of 254 nm. For the acetonide-protected caffeic, using the same column and mobile phase acetonitrile and phosphoric acid (70:30), with an injection volume of 5 μL. The compound is detected at a wavelength of 324 nm.

Hydrogel curing. The hydrogels were cured from solutions of PEG1k$(SH)_2$, vinyl-functional dendrimers and DMPA, irradiated for 4-6 hours with a UV lamp. A UV darkroom Vilber CN-15.LC was used, with a total intensity of 30 W at 365 nm.

The compounds were dissolved in a THF:MeOH (1:2) mixture, argon bubbled for a few seconds and introduced into several Teflon plugs before exposure to UV light.

Swelling studies. The swelling assays were done exposing the hydrogels to a distilled water or buffer solution for several days. All gels were tested in duplicate. The swelling degree was calculated with the following equation:

$$SD\% = ((W-W_D) / W) * 100$$

where SD% is the percentage of swelling, W is the weight of the swollen gel and W_D is the dry gel mass after washing.

Crosslinking studies. The crosslinking percentage was calculated using the following equation:

$$CD(\%) = (1- (M_{UV}-M_W / M_{UV}) * 100$$

where CD is the crosslinking degree, W_{UV} is the dry mass after UV and M_W is the dry mass after washing. All measurements were performed in duplicate.

Scanning Electron Microscopy. Hydrogels were washed in Milloning's solution and fixed in Milloning's solution containing 2% glutaraldehyde overnight. After fixation, samples were washed twice in Milloning's solution and dehydrated through an ethanol gradient (30, 50, 70, 95, 100; 7 min for each concentration), and placed in anhydrous acetone solution. Then, samples were critical-point dried using a Polaron CPD7501 critical-point drying system. After that, hydrogels were

cut in two pieces, placed on pin mount specimen holders (top and bottom) using double-sided carbon tap and coated with 200 Å gold-palladium using a Polaron E5400. SEM was performed in a STEM JSM-IT500 LV (JEOL).

Figures.

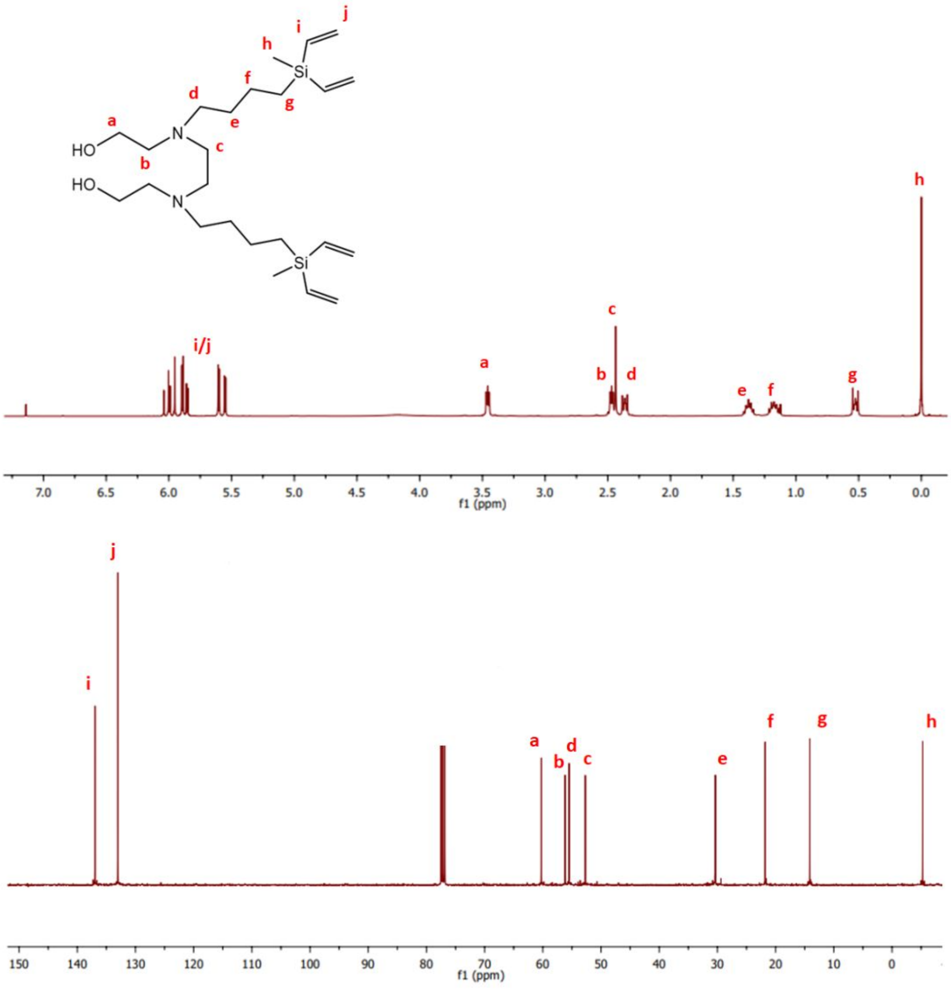

Figure S1. ^{1}H and ^{13}C NMR spectra of dendrimer N_2O_2-G1V_4 **(1)** in $CDCl_3$.

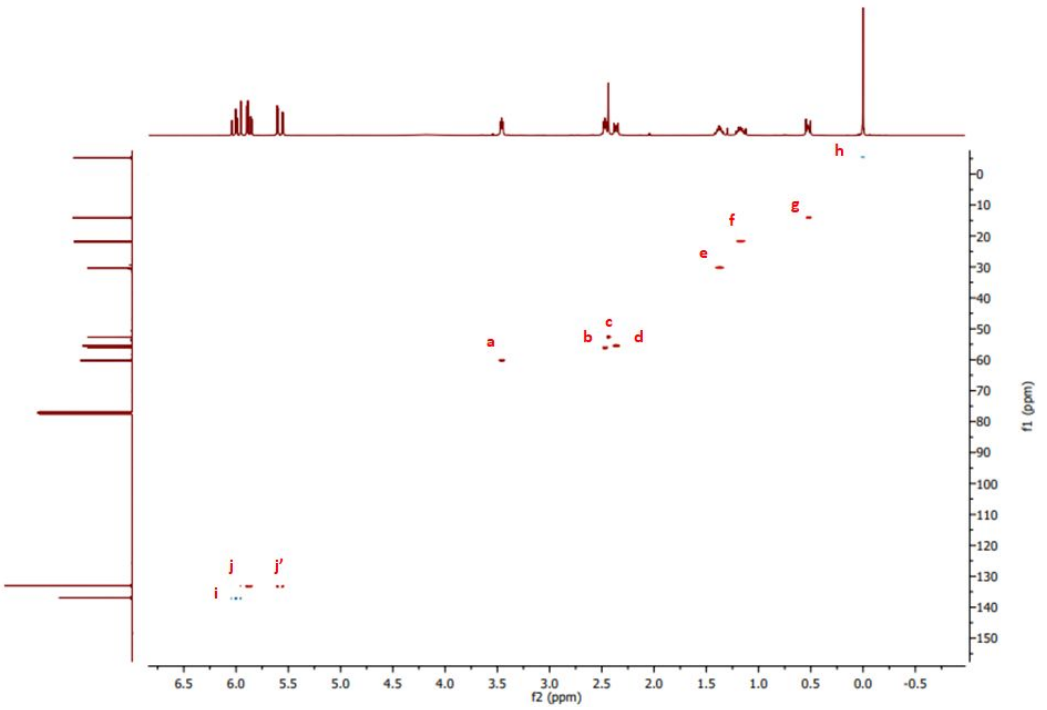

Figure S2. ^{1}H-^{13}C HSQC spectra of dendrimer N_2O_2-G1V_4 **(1)** in CDCl3.

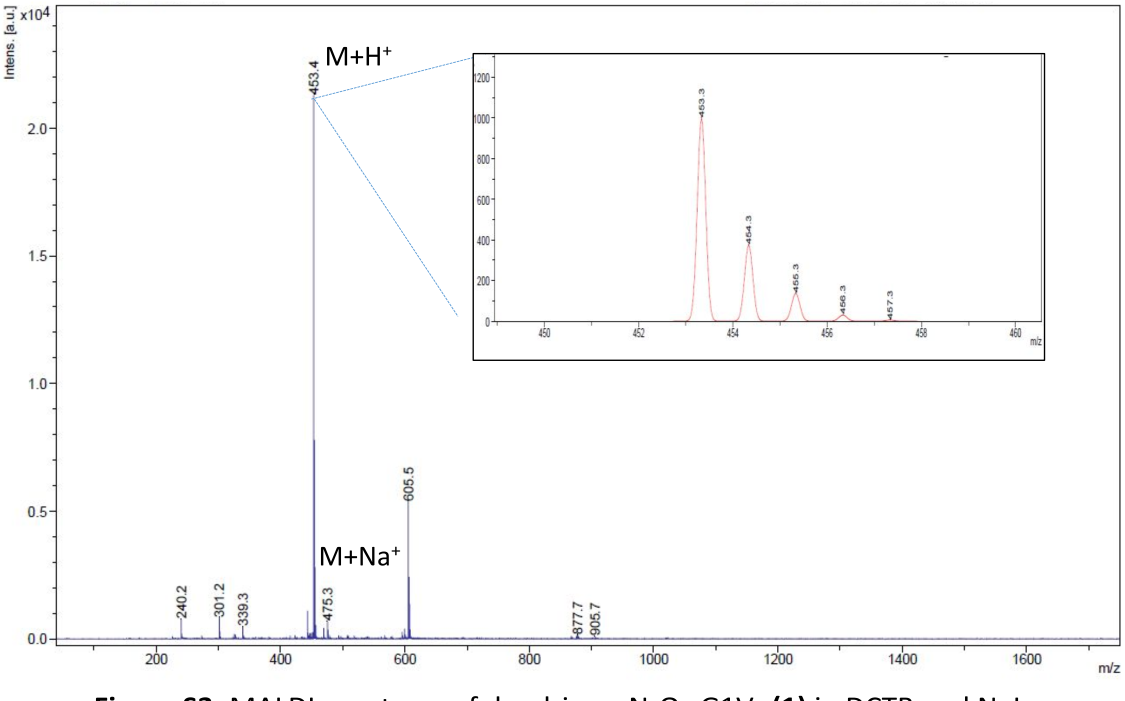

Figure S3. MALDI spectrum of dendrimer N_2O_2-G1V_4 **(1)** in DCTB and NaI.

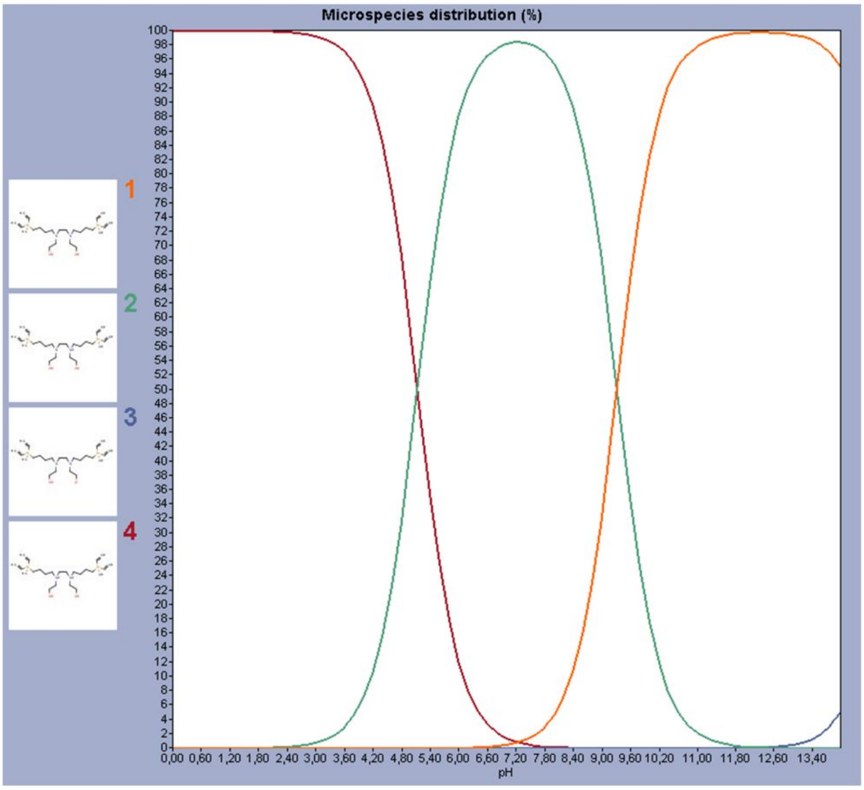

Figure S4. Prediction of pK_a values (5,14 and 9,33 for the two nitrogen atoms) and microspecies distribution for dendrimer **1**, as calculated through MarvinSketch 22.7. At pH 7.4, the predominant specie presents one nitrogen protonated.

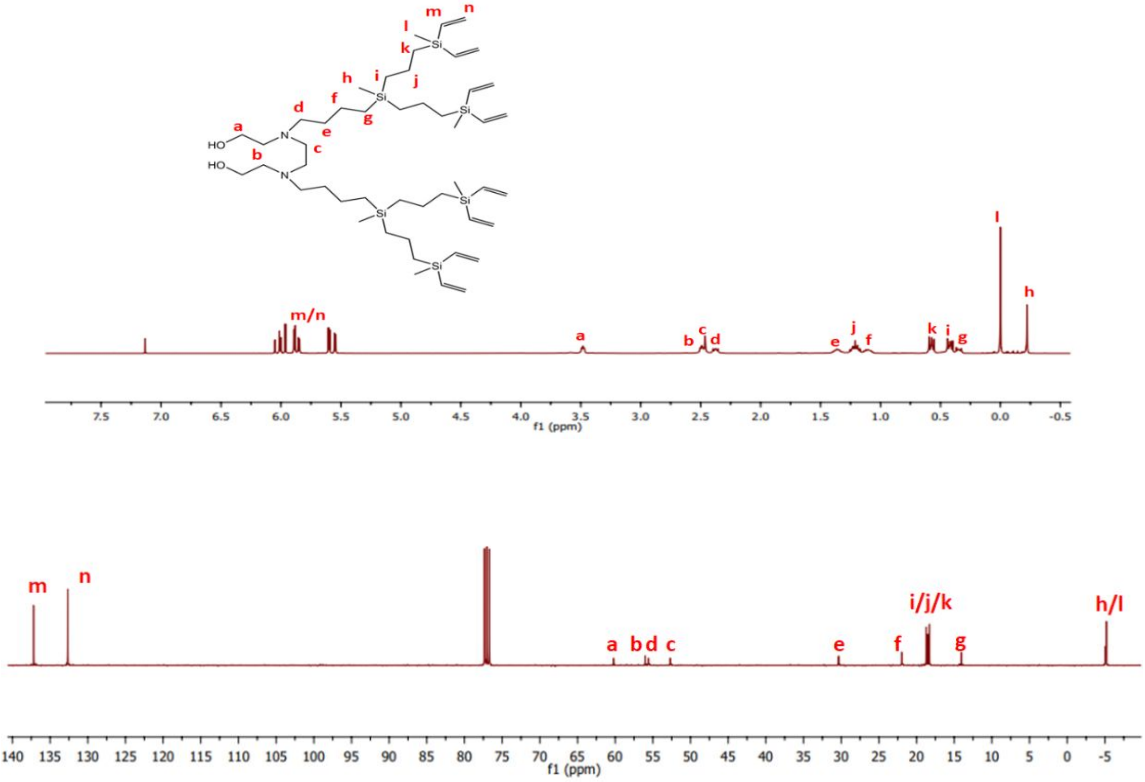

Figure S5. ^{1}H and ^{13}C NMR spectra of dendrimer N_2O_2-G2V_8 **(2)** in $CDCl_3$.

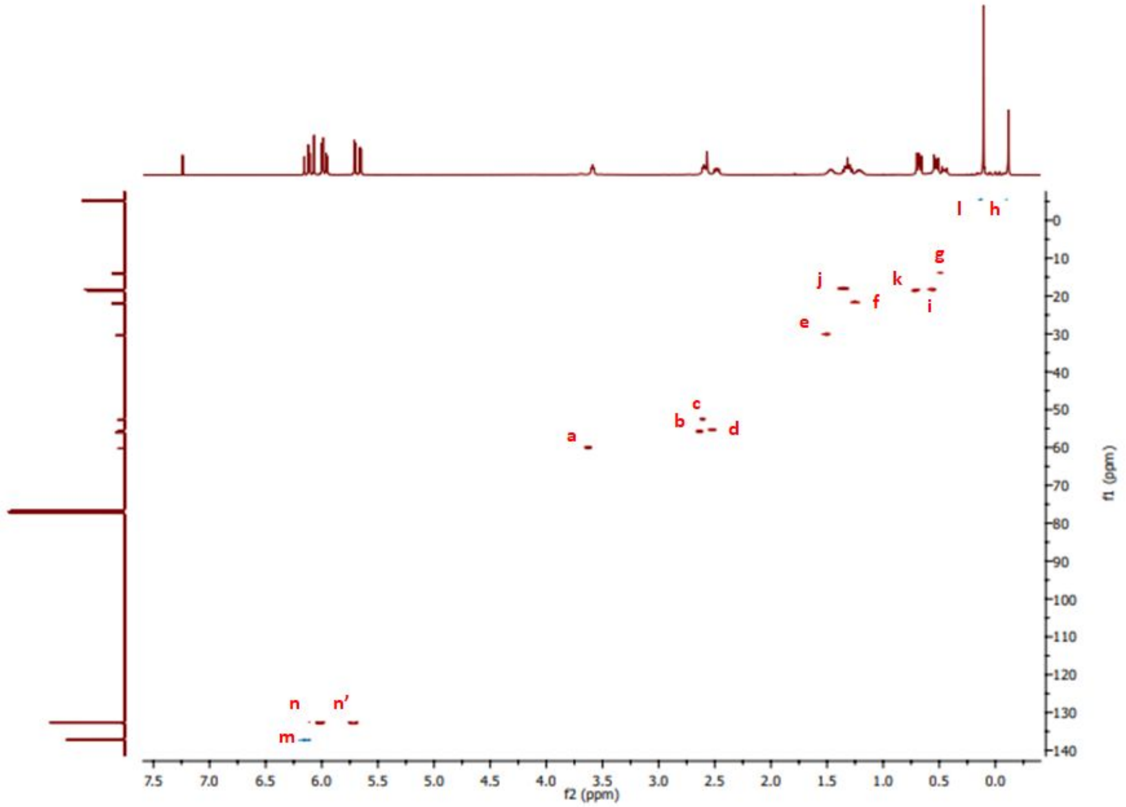

Figure S6. ^{1}H-^{13}C HSQC spectra of dendrimer N_2O_2-G2V_8 **(2)** in CDCl3.

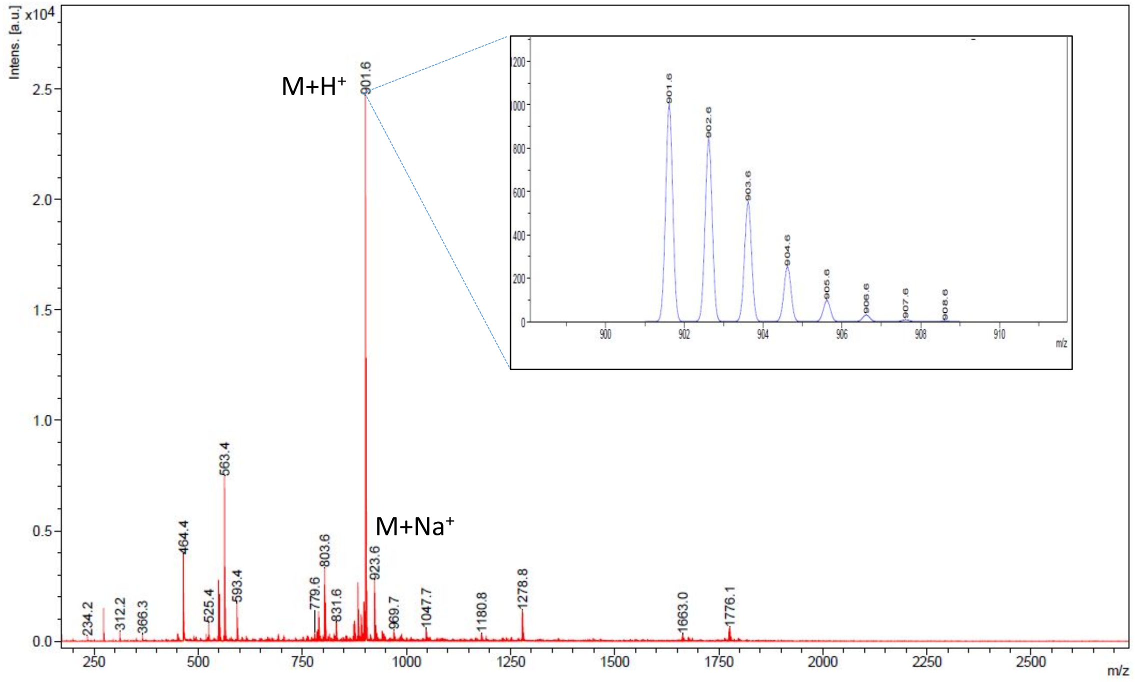

Figure S7. MALDI spectra of dendrimer N_2O_2-G2V_8 **(2)** in DCTB and NaI.

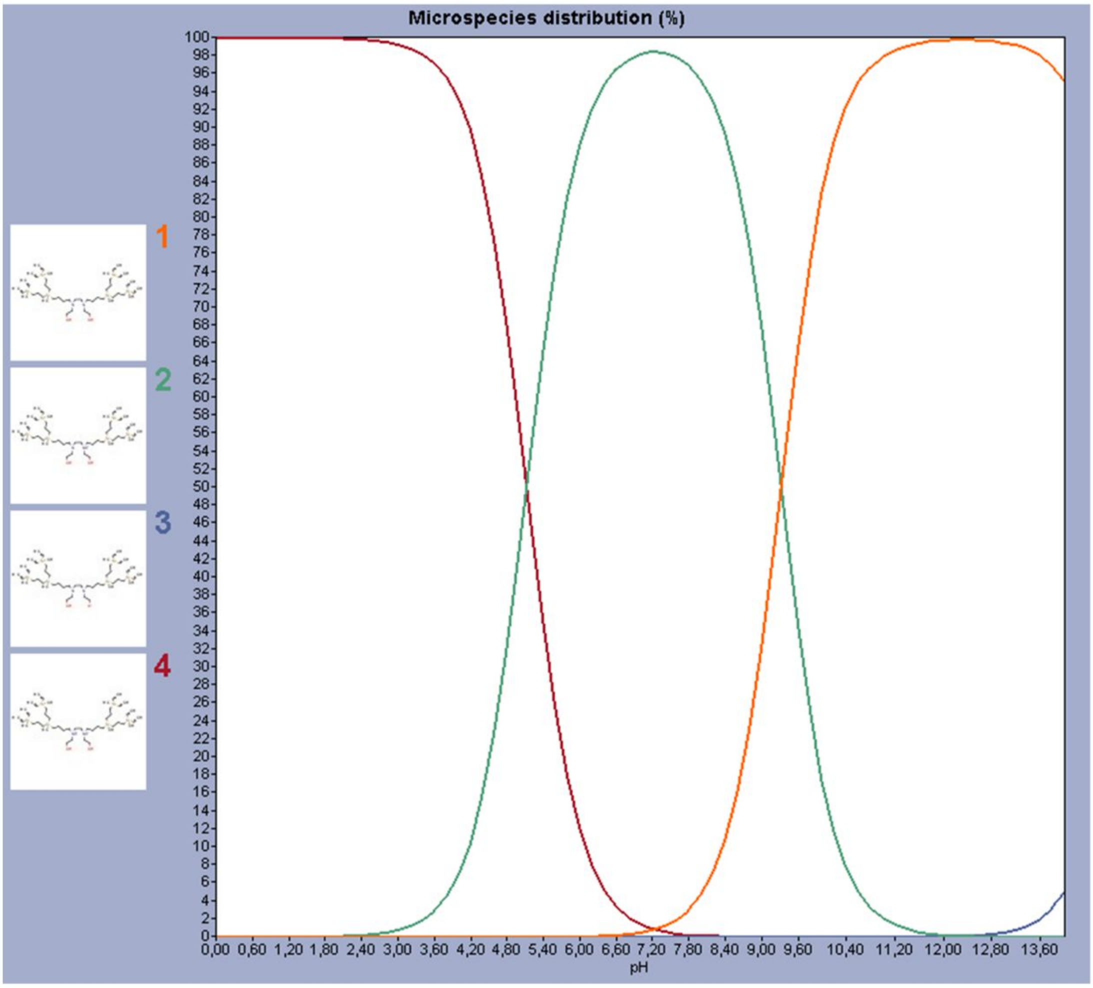

Figure S8. Prediction of pK_a values (5,14 and 9,33 for the two nitrogen atoms) and microspecies distribution for dendrimer **2**, as calculated through MarvinSketch 22.7. At pH 7.4, the predominant specie presents one nitrogen protonated.

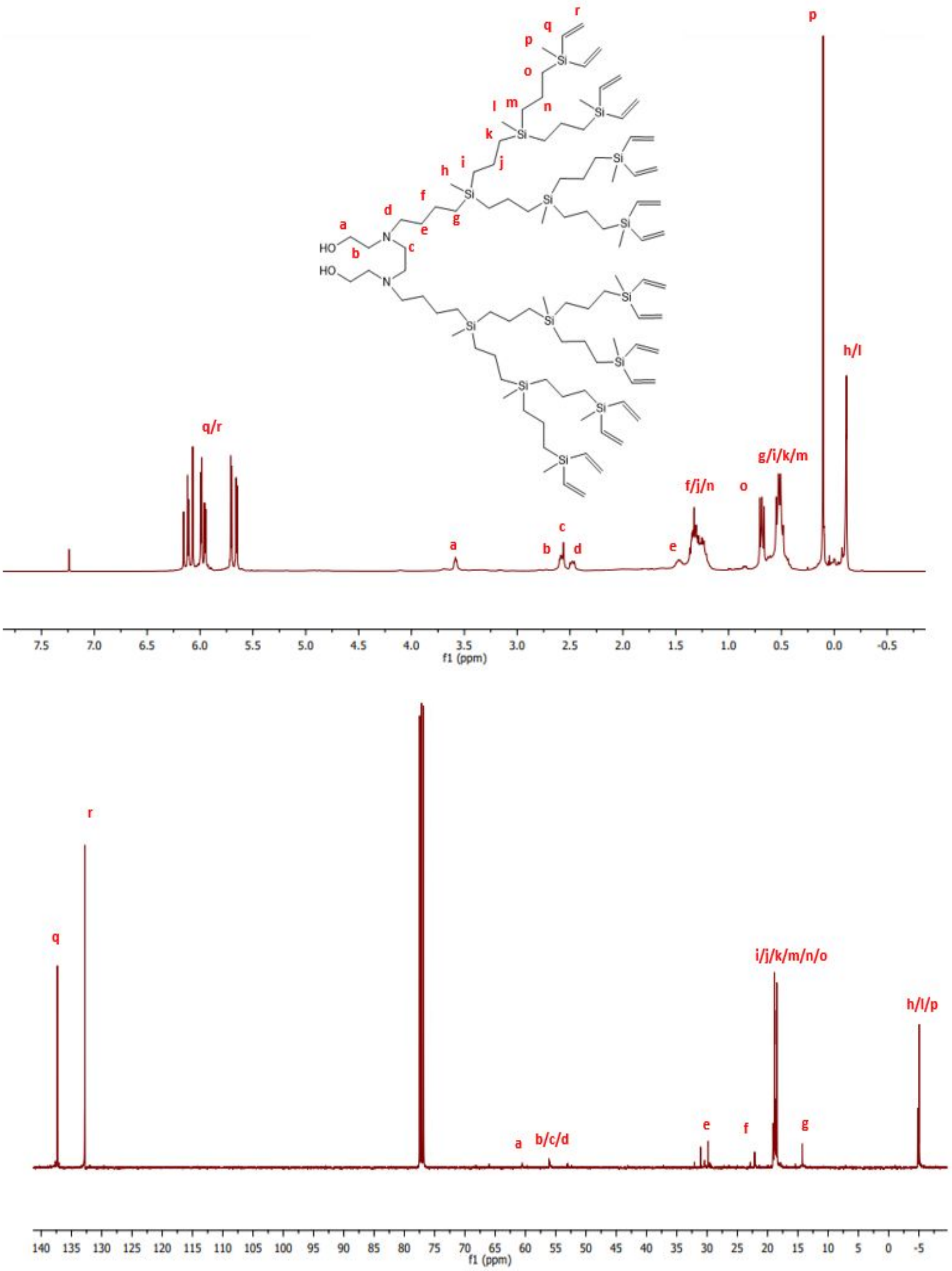

Figure S9. ^{1}H and ^{13}C NMR spectra of dendrimer N_2O_2-G3V_{16} **(3)** in $CDCl_3$.

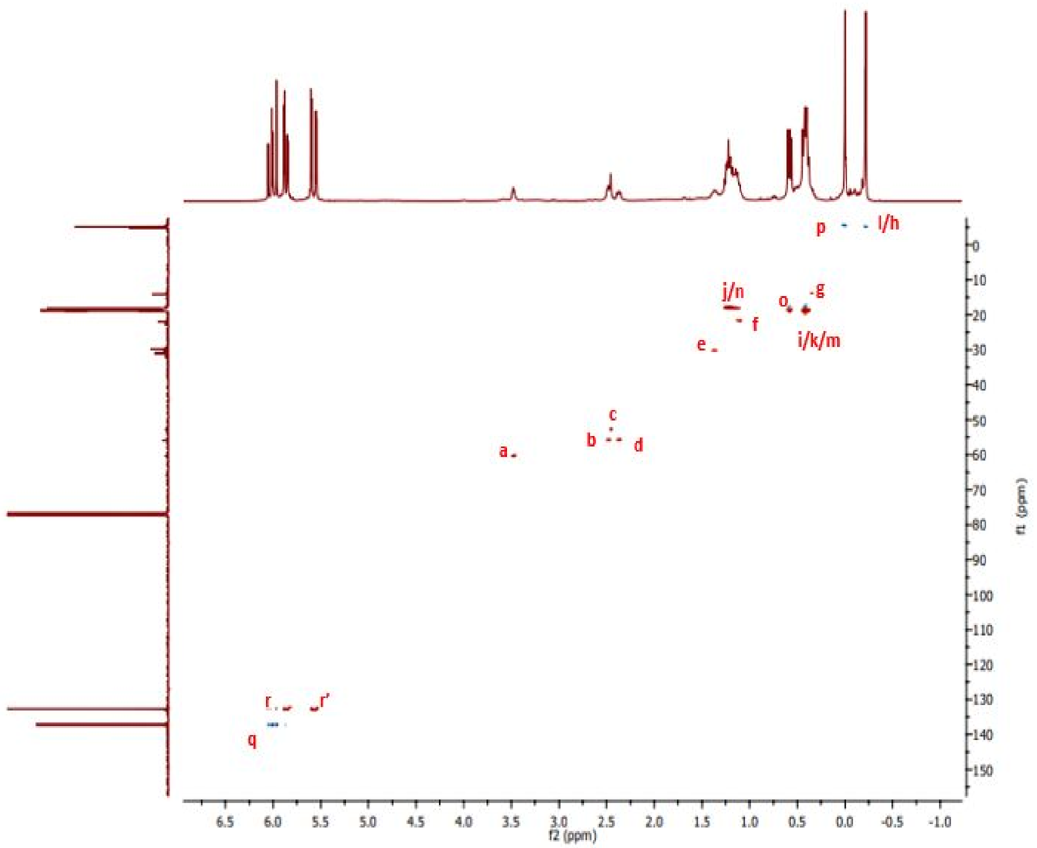

Figure S10. ^{1}H-^{13}C HSQC spectra of dendrimer N_2O_2-G3V_{16} **(3)** in CDCl3.

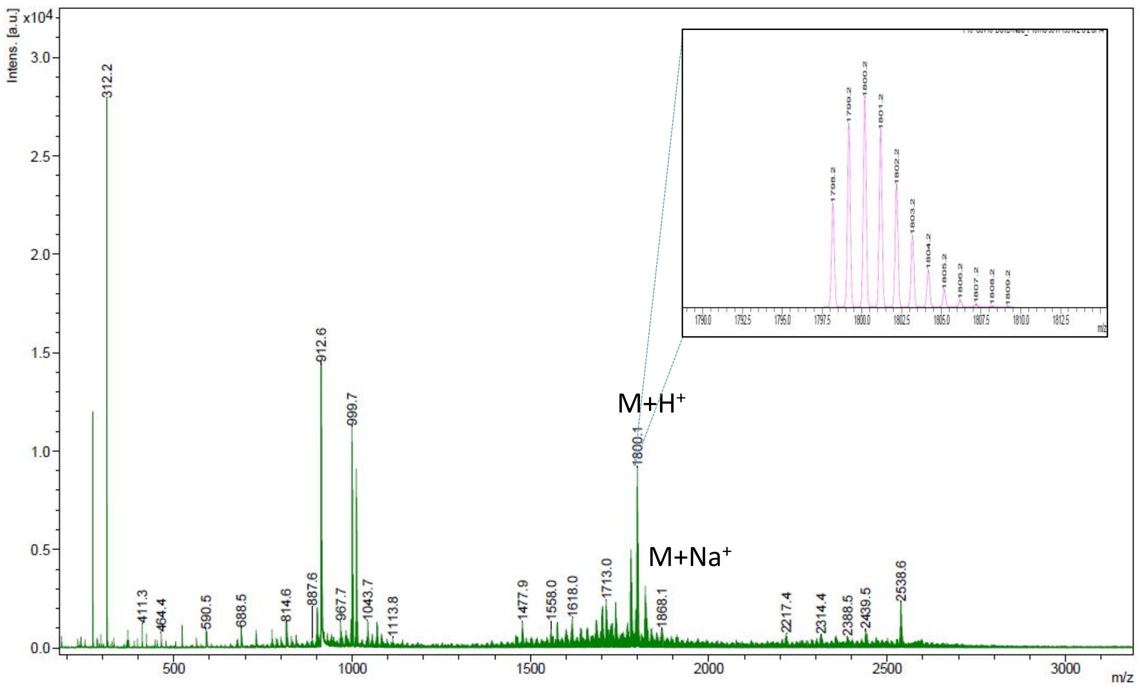

Figure S11. MALDI spectra of dendrimer N_2O_2-G3V_{16} **(3)** in DCTB and NaI.

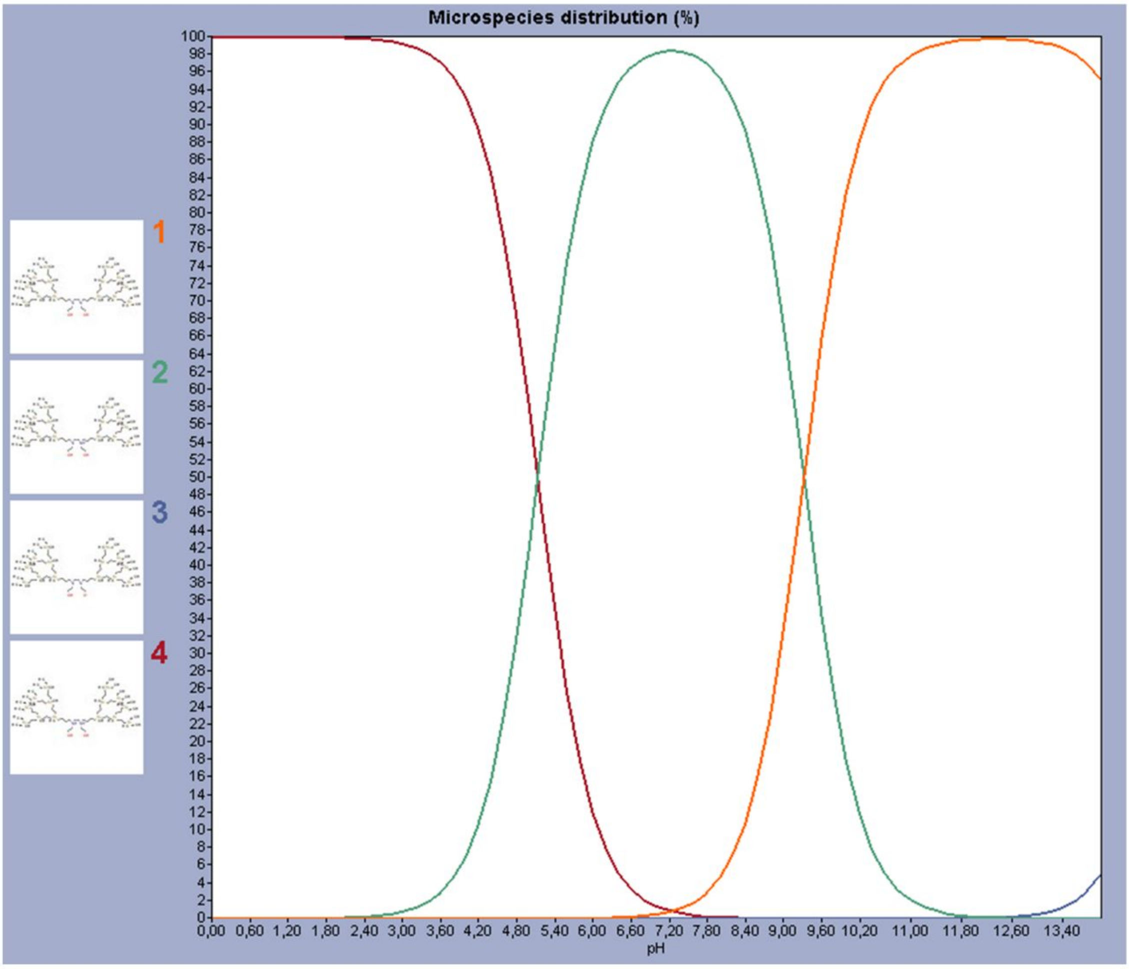

Figure S12. Prediction of pK_a values (5,14 and 9,33 for the two nitrogen atoms) and microspecies distribution for dendrimer **3**, as calculated through MarvinSketch 22.7. At pH 7.4, the predominant specie presents one nitrogen protonated.

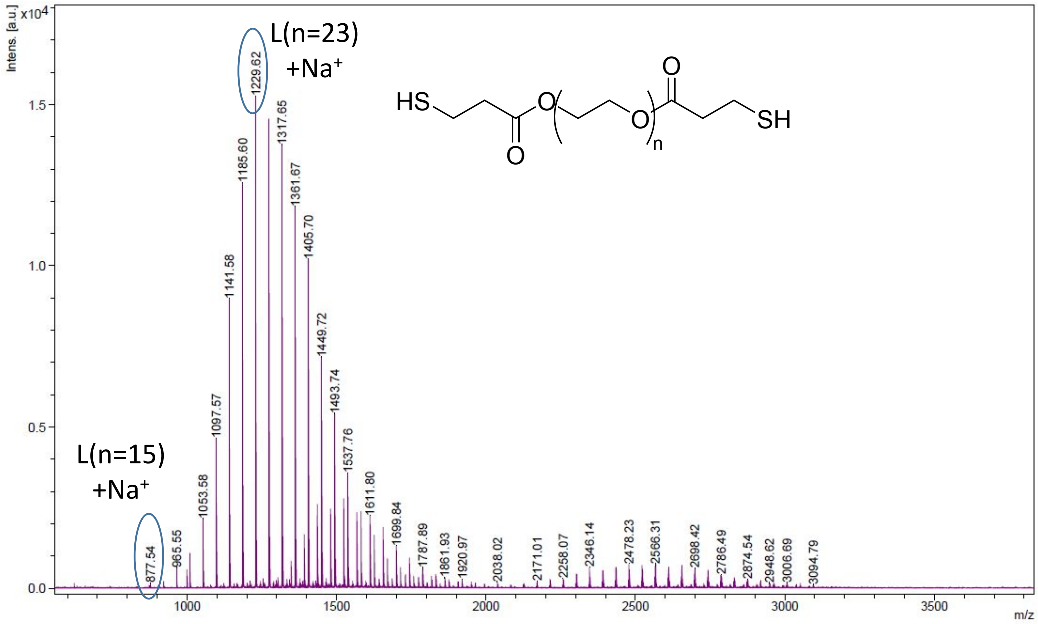

Figure S13. MALDI spectra of PEG1k(SH)$_2$ (**7**) in DCTB and NaI.

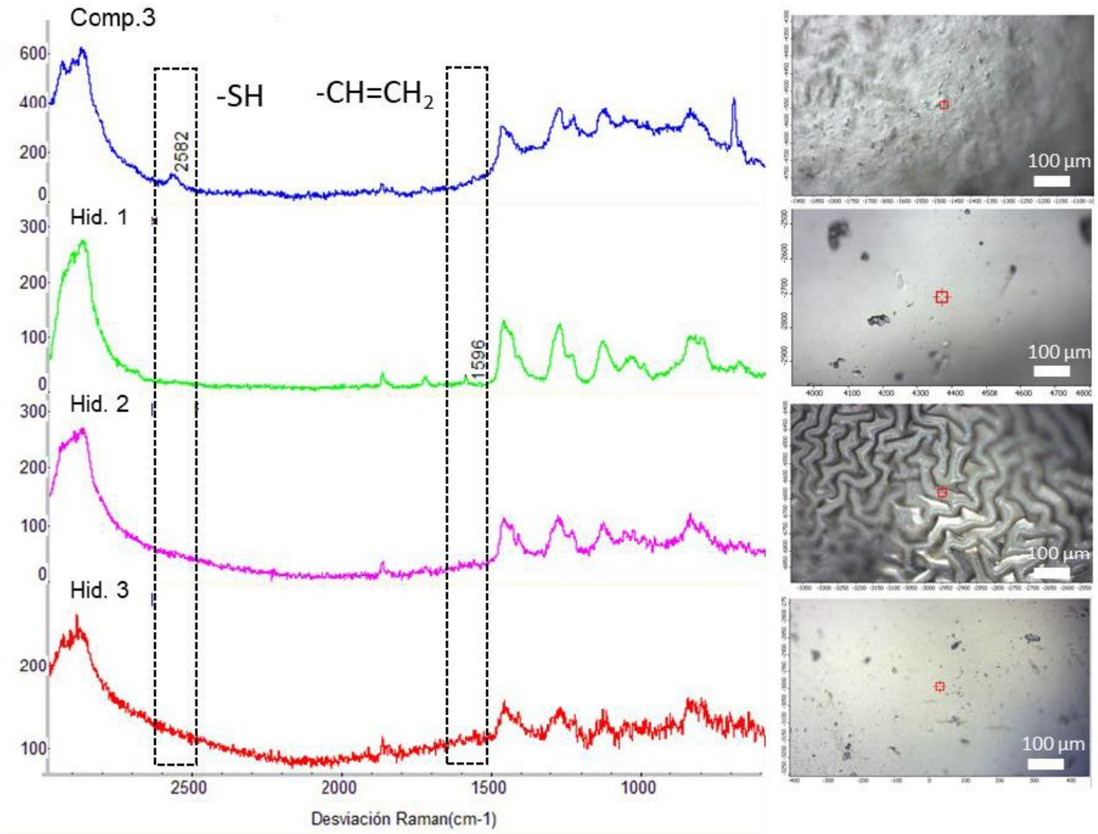

Figure S14. RAMAN-confocal spectra of polymeric precursor PEG1k$(SH)_2$ (**7**, A) and STE hydrogels **H4** (B), **H1** (C) and **H2** (D).

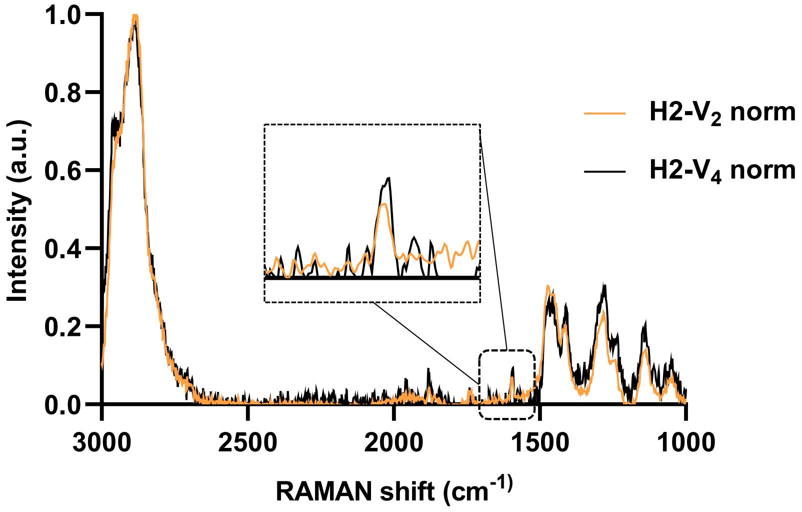

Figure S15. RAMAN-confocal spectra of OSTE hydrogels **H2-V**$_2$ and **H2-V**$_4$.

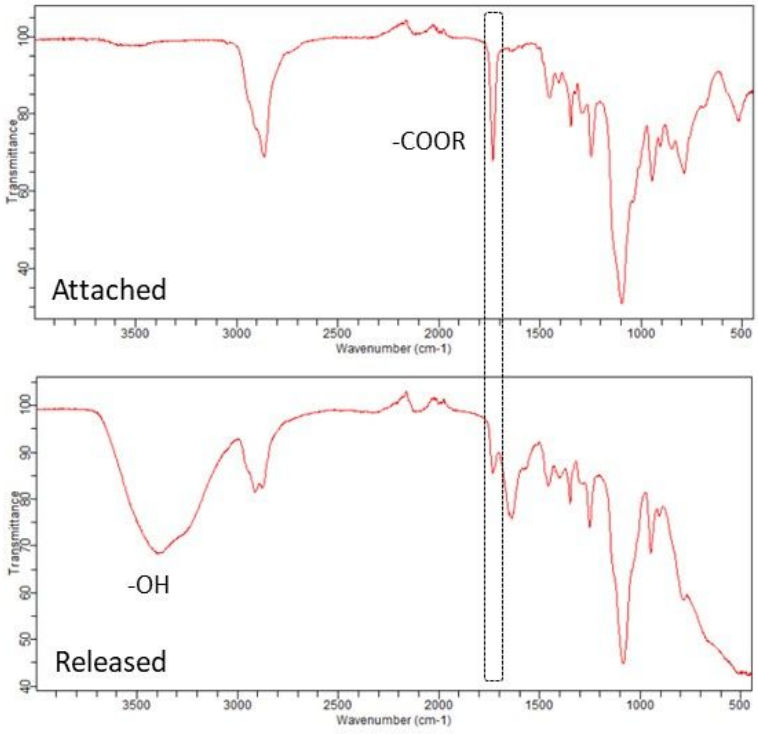

Figure S16. FT-IR spectra of Hy[(N_2(OIbu)$_2$-G2V_6)x(P)]V_2 (**Ibu-H2-V_2**) before (top) and after (bottom) exposure to FBS-containing solution. The peak corresponding to ester bonds is highlighted.

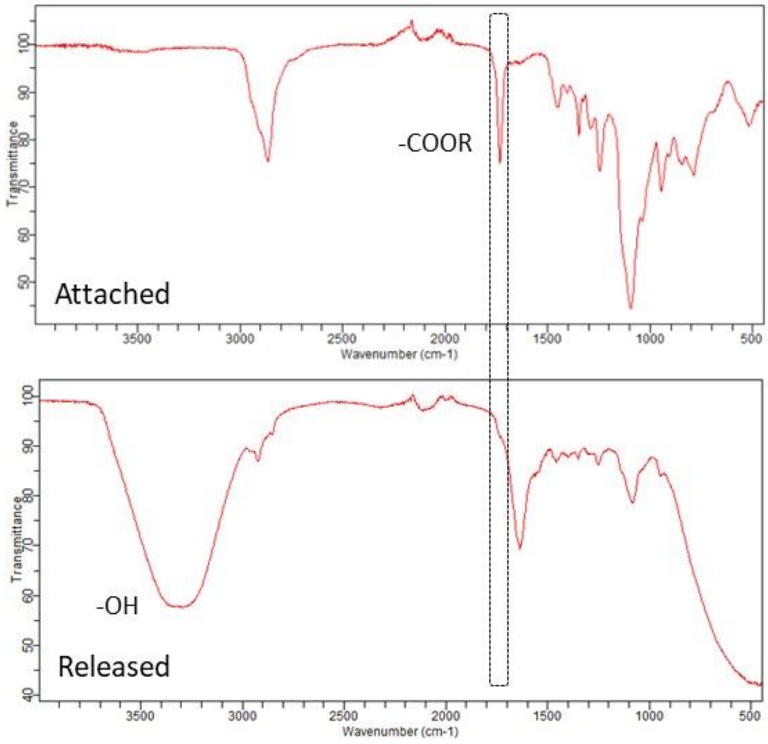

Figure S17. FT-IR spectra of Hy[(N_2(OCaf)$_2$-G2V_6)x(P)]V_2 (**Caf-H2-V_2**) before (top) and after (bottom) exposure to FBS-containing solution. The peak corresponding to ester bonds is highlighted.

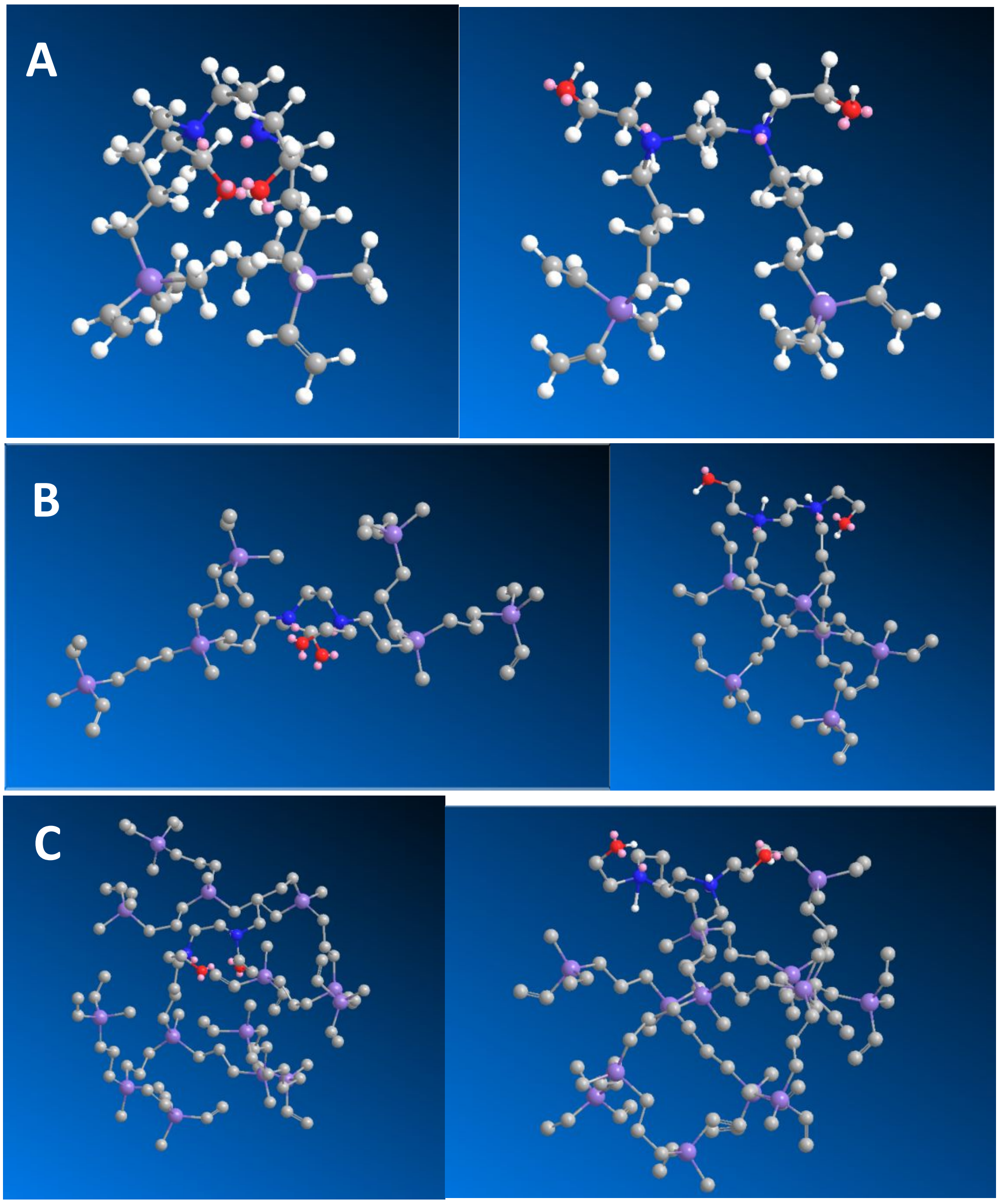

Figure S18. Snapshots from 3D spatial arrangement of: **A)** Dendrimer **1** with the two nitrogen atoms deprotonated (left) or protonated (right). For deprotonated conditions: Distance (N,N): 2.851 Å, Distance (O,O): 5.559 Å. For protonated conditions: Distance (N,N): 3.906 Å, Distance (O,O): 9.827 Å. **B)** Dendrimer **2** with the two nitrogen atoms deprotonated (left) or protonated (right). For deprotonated conditions: Distance (N,N): 2.960 Å, Distance (O,O): 5.214 Å. For protonated conditions: Distance (N,N): 4.163 Å, Distance (O,O): 7.659 Å. **C)** Dendrimer **3** with the two nitrogen atoms deprotonated (left) or protonated (right). For deprotonated conditions: Distance (N,N): 3.186 Å, Distance (O,O): 5.842 Å. For protonated conditions: Distance (N,N): 3.921 Å, Distance (O,O): 7.591 Å. All structures have been optimized according to the following steps: [Job 1 (Minimize Energy to Minimum RMS Gradient of 0.010) + Job 2 (Molecular Dynamics. Step Interval: 2.0 fs. Frame Interval: 10 fs. Terminate After: 10000 steps. Heating/Cooling Rate: 1.000 Kcal/atom/ps. Target Temperature: 300 K)].

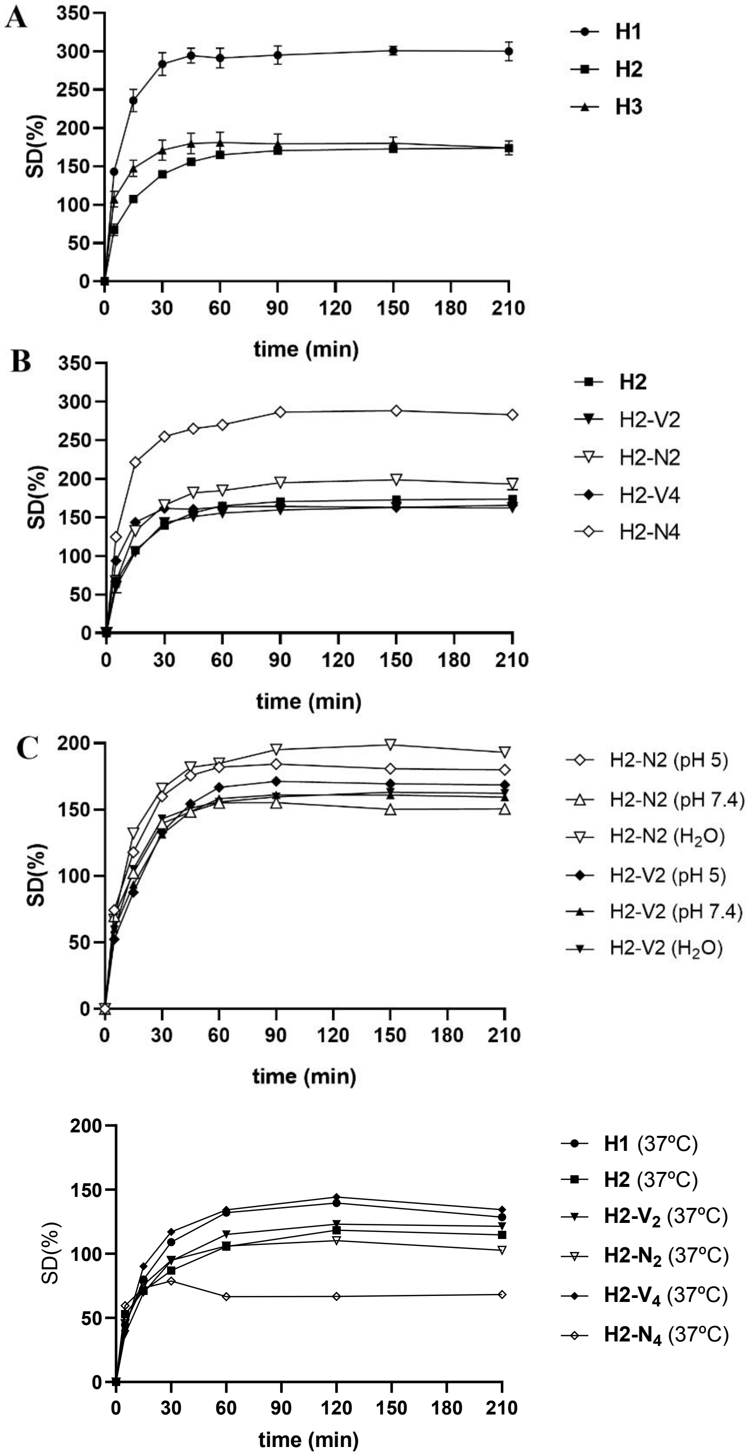

Figure S19. Swelling degree in water at 25 °C for (A) STE hydrogels **H1**, **H2** and **H3**; (B) OSTE hydrogels **H2-V$_2$**, **H2-V$_4$**, **H2-N$_2$** and **H2-N$_4$**; (C) OSTE hydrogels **H2-V$_2$** and **H2-N$_2$** in H_2O and in buffers at pH 5.5 and 7.4. Results are the average of three different independent experiments. D. Swelling degree in water at 37 °C for STE hydrogels **H1** and **H2**, and OSTE hydrogels **H2-V$_2$, H2-V$_4$, H2-N$_2$** and **H2-N$_4$**.

- Experiment #1 (Fig. S19A): Selected hydrogels were immersed in water at 25°C and both their diameter and thickness were measured over time. All hydrogels exhibit a similar swelling pattern: the SD% increased exponentially and reached a plateau after 60 minutes. For STE hydrogels **H1** and **H3**, the SD values decreased when increasing the dendrimer generation and the corresponding lipophilicity, from 300% in **H1** to 180% in **H3** (**Fig. 3.A**). Despite that the three STE hydrogels exhibited similar CD% values in the range 80-90%, the swelling was surprisingly high for **H1**, probably due to the higher hydrophilicity of the precursor dendrimer, while **H2** and **H3** exhibited a similar behaviour, due to the high lipophilicity of both dendrimers **2** and **3** that prevent a comfortable loading of water within the pores of the hydrogels.
- Experiment #2 (Fig. S19B): For OSTE hydrogels, the vinyl-functional hydrogels **H2-V$_2$** and **H2-V$_4$** exhibited the same swelling than the STE counterpart **H2**, around 150%. Surprisingly, the different CD% and the presence of pendant vinyl groups did not affect the swelling of the network. However, when functionalized with ammonium groups, the SD increased to

200% for **H2-N$_2$** and 290% for **H2-N$_4$**. The electrostatic repulsion produced by close cationic groups favour the increase in swelling degree.

- Experiment #3 (Fig. S19C): To check the impact of pH, the SD% of selected hydrogels (**H2-V$_2$** and **H2-N$_2$**) was studied in two different buffer solutions at pH 5.5 and 7.4, compared with unbuffered water. Subtle changes occur for **H2-V$_2$** under the three different conditions; however, a slightly higher SD is observed at pH 5.5, when more nitrogen atoms at the dendrimers core are protonated. A more pronounced effect is observed for **H2-N$_2$**, with pendant ammonium groups. A 30% higher swelling is found at pH 5.5 compared to pH 7.4, due to the repulsion of the cationic groups. The surprisingly higher swelling in unbuffered water is explained by the partial release of HCl from the ammonium groups, which lead to an overall solution pH of 5 where all nitrogen atoms in the dendrimers core are protonated.
- Experiment #4 (Fig. S19D): Additionally, we explored the temperature-responsive behaviour of our hydrogels by analysing SD% at 37°C. All hydrogels exhibited a significantly lower swelling at this temperature. The maximum SD% was reached after 2 h and then it started decreasing slowly. For

example, STE hydrogels **H1** and **H2** reached a maximum SD of 140% and 119% respectively, which is 64% and 31% reduction from the swelling at 25°C. This effect is especially relevant for ammonium-functional hydrogels **H2-N$_2$** and **H2-N$_4$**. At 37°C, they underwent a 45% and 77% reduction of swelling respectively, compared to 25°C. This temperature-dependent behaviour must be also considered when using these hydrogels for drug delivery purposes.
